# Influence of Seasonal Variability in Flux Attenuation on Global Organic Carbon Fluxes and Nutrient Distributions

**DOI:** 10.1029/2021GB007101

**Published:** 2022-02-07

**Authors:** Francisco de Melo Viríssimo, Adrian P. Martin, Stephanie A. Henson

**Affiliations:** ^1^ National Oceanography Centre Southampton UK

**Keywords:** biological carbon pump, particle flux attenuation, particulate organic carbon (POC), transfer efficiency, seasonal variability, carbon sequestration

## Abstract

The biological carbon pump is a key component of the marine carbon cycle. This surface‐to‐deep flux of carbon is usually assumed to follow a simple power law function, which imposes that the surface export flux is attenuated throughout subsurface waters at a rate dictated by the parameterization exponent. This flux attenuation exponent is widely assumed as constant. However, there is increasing evidence that the flux attenuation varies both spatially and seasonally. While the former has received some attention, the consequences of the latter have not been explored. Here we aim to fill the gap with a theoretical study of how seasonal changes in both flux attenuation and sinking speed affect nutrient distributions and carbon fluxes. Using a global ocean‐biogeochemical model that represents detritus explicitly, we look at different scenarios for how these varies seasonally, particularly the relative “phase” with respect to solar radiation and the “strength” of seasonality. We show that the sole presence of seasonality in the model‐imposed flux attenuation and sinking speed leads to a greater transfer efficiency compared to the non‐seasonal flux attenuation scenario, resulting in an increase of over 140% in some cases when the amplitude of the seasonality imposed is 60% of the non‐seasonal base value. This work highlights the importance of the feedback taking place between the seasonally varying flux attenuation, sinking speed and other processes, suggesting that the assumption of constant‐in‐time flux attenuation and sinking speed might underestimate how much carbon is sequestered by the biological carbon pump.

## Key Points

- The inclusion of seasonal variability in the flux attenuation parameter is found to increase the particulate organic carbon transfer to depth
- The increase observed is sensitive to the relative phase between the flux attenuation and the solar radiation
- A constant‐in‐time flux attenuation might underestimate how much carbon is sequestered by the ocean

## 1 Introduction

The carbon cycle is one of the most important chemical cycles in the Earth system, with much of it taking place in the marine environment. An important contribution to this cycle is given by the ocean's biological carbon pump (hereafter BCP; Volk & Hoffert, 2013). The BCP works by transferring organic carbon fixed via photosynthesis from the upper ocean to the deep interior, often in the form of sinking particles, or particulate organic carbon (POC). This biota‐driven “pump” mechanism starts with only a small proportion of primary production (PP) leaving the epipelagic zone (upper 100–200 m) as POC. After this *export* of POC, an even smaller quantity of organic material survives the mesopelagic environment (200–1,000 m) and reaches the very deep ocean, where it can remain for hundreds of years. The fraction of the export flux entering the deep ocean (here considered to be ocean region below 1,000 m depth) is quantified by the transfer efficiency (TE). Several factors affect the transfer efficiency, each of them varying seasonally and regionally. Key among these factors are both remineralization and sinking of dead organic material, which is in turn related to other components such as plankton community structure (Bach et al., 2019; Ikenoue et al., 2019), heterotrophic organisms (Zakem & Levine, 2019) and biogenic minerals (Armstrong et al., 2001; Klaas & Archer, 2002), as well as to temperature and oxygen distributions (Cavan et al., 2017). Combined, the remineralization and sinking effects dictate how the POC flux is attenuated throughout the mesopelagic ocean.

Despite the well‐known seasonal and regional influences of the aforementioned mechanisms on remineralization and sinking, these are widely assumed as constant in time and space. This is partly motivated by earlier studies such as Martin et al. (1987), which proposed simple parameterizations (see also Methods) for the flux and attenuation of organic matter in the open ocean, which has since been widely used both by observationalists and modelers in the field. Over the years, however, the hypothesis of a constant and spatially uniform remineralization profile has been challenged, based on observed temporal and regional variability tendencies in the flux attenuation profile (Buesseler & Boyd, 2009; Francois et al., 2002).

Evidence of variability in the flux attenuation emerged from sediment‐trap samples collected over the last few decades. A series of such sediment‐trap observations (Berelson, 2001; Buesseler et al., 2007; Conte et al., 2001; Francois et al., 2002; Lutz et al., 2002) showed a flux attenuation parameter (estimated from Equation 1 in Methods) varying spatially from 0.5 to 2.0 around the globe (as per Kwon et al., 2009, compiled from the references above). This variability is important because changes in the flux attenuation can have a significant impact on the distribution of nutrients in the global ocean and on the carbon cycle. This was evidenced in a study by Yamanaka and Tajika (1996), as they showed that varying the flux attenuation parameter (as per Equation 1 in Methods) globally from 0.9 to 2.0 led to an atmospheric partial pressure of carbon dioxide (CO_2_) change of about 100 ppm. Later, Kwon et al. (2009) showed that even smaller variations in the remineralization profile can change substantially the global distribution of nutrients.

In the context of POC and nutrient fluxes, variations in the flux attenuation can be linked to a variation in TE (and vice versa)—for instance through Equations 1 and 2 shown in Methods. In some cases, TE is used preferentially to the flux attenuation, as it requires fewer assumptions and is less sensitive to the choice of reference depth and shape of profile. However, for situations where there are observations of the POC flux at more than 2 depths, then a flux attenuation parameter frequently used. For this purpose, some studies may use an exponential or a power law such as Equation 1 in Methods.

Since evidence of variability in the flux attenuation emerged, the case for spatial variability has received the most attention. In terms of global spatial patterns in attenuation and TE, Henson et al. (2012) found that TE is high at low latitudes and low at high latitudes, while the flux attenuation (as per Equation 2 in Methods) is high at high latitudes and low at low latitudes, a result that aligns with later works from Guidi et al. (2015) and Mouw et al. (2016). Contradictory to that, Marsay et al. (2015) presented data from North Atlantic and North Pacific which show that the spatial variability in the flux profiles can be largely explained by temperature, with warmer waters, typically in lower latitudes, being correlated with the shallowest remineralization, meaning higher values of the flux attenuation parameter and lower TE (see Equation 2). Weber et al. (2016) used diagnostics from a data‐constrained ocean circulation model to obtain deep ocean particle flux profiles, from which they found a global pattern of TE to 1,000 m that is higher at high latitudes than in subtropical gyres, and assumes intermediate values in the tropics—a result that agrees with the aforementioned work of Marsay et al. (2015).

Several studies have also found a substantial seasonal variability in export and TE, as well as in the average sinking speed of POC, which is widely accepted as one of the key contributors to both TE and flux attenuation (Middelburg, 2019; Weber et al., 2016)—see also Equation 3. When exploring how the BCP efficiency is related to global‐scale environmental parameters and the seasonality of net PP, Lutz et al. (2007) found a seasonal pattern of export flux, with the sinking fraction of net PP during intervals of bloom production being typically half that of other seasons. A strong seasonal variability in the average sinking velocity of particles was also found in a North Atlantic‐based study by Villa‐Alfageme et al. (2016), possibly related to changes in the epipelagic community structure in that location. In relation to seasonality in transfer to depth, although the seasonality in deep ocean fluxes is well studied (e.g., Hartman et al., 2012; Muller‐Karger et al., 2019; Smith et al., 2018, all showing a high seasonality in deep POC fluxes), the export flux is not often seasonally resolved, and therefore seasonal variability in TE is poorly known. The few exceptions include: both HOT (University of Hawaii, 2021) and BATS (Conte et al., 2019) stations, where monthly ship‐board sampling has been done; the PAP station (Bol et al., 2018), where autonomous vehicles have been deployed for a 1‐year period; and parts of the Norwegian Sea (Dall’Olmo & Mork, 2014) and the Subtropical Atlantic (Estapa et al., 2019), where autonomous platforms such as Bio‐Argo floats have been used. In particular, recent work by Bol et al. (2018) presented a full‐year data set of backscatter‐derived POC data measured by seagliders in the North Atlantic PAP site, at daily resolution, showing that the POC export to the mesopelagic zone displays a strong seasonality with deepest remineralization occurring during winter and shallowest during late spring and summer.

Despite evidence that it exists, we still lack an understanding of the impact of seasonal variability in TE. To exemplify that, an important question that remains unanswered is what is the impact of seasonal variation in the flux attenuation and sinking speed when these lead or lag the well‐known seasonal variability in surface primary production. This is important since, for instance, periods of low sinking speed coinciding with the spring bloom might result in a less efficient POC transfer to depth on an annual timescale than if the maximum sinking speed (or weakest attenuation) occurred during the bloom. This lack of understanding is in spite of the phenomenon often being present in models, as many of them simulate POC explicitly. This means that POC is represented as a tracer that is dependent on other dynamically resolved tracers such as phytoplankton, zooplankton and nutrient distribution (see for instance Equation 27 in Kriest et al., 2010). This implies a time variability in the detritus profiles and leads to a seasonal variation in the diagnosed fluxes (given by the sinking speed times the detritus concentration) and consequently in TE as well.

An obstacle to tackling this topic is that most measurements of flux attenuation profiles are made from sinking particle flux observations taken at specific points in time. These are usually in spring and summer, such that we currently lack a clear picture of the full seasonal cycle in the flux attenuation and its relation to the seasonal cycle in surface primary production. Here we address this by testing how seasonal variability in both flux attenuation and sinking speed in a model that represents detritus explicitly can impact global POC fluxes and nutrient distributions, and we do this by using a global biogeochemical model modified to incorporate simplified representations of seasonally varying flux attenuation parameter and sinking speed that allow us to explore the possible range of seasonal variability.

To the best of our knowledge, this is the first work that explicitly looks at the consequences of seasonally varying flux attenuation for both export and transfer of POC to depth, as well as its impact on the distribution of nutrients in the ocean. In this study, this is done by comparing results obtained using constant and uniform flux attenuation and sinking speed to those found using different seasonality patterns which vary both in strength and phase. The latter is taken into account by varying the phase of the seasonal variation in the flux attenuation with respect to phytoplankton growth and solar radiation (see Methods for details).

This paper is divided as follows. In Section 2 we present the model used, as well as the parameters and variables that are relevant to the study. The set of experiments to be performed are also presented and discussed in this section. Results for nutrient distributions, primary production, POC export and transfer to depth are presented in Section 3. These results are discussed in Section 4 in the light of the influence of seasonality on the BCP. Conclusions and further directions are provided in Section 5.

## 2 Methods

The influence of remineralization on the POC transfer to depth is often estimated through a power law curve, as proposed by Martin et al. (1987), equating the POC flux *F*(*z*) at depth *z* with the POC flux $F_{z_{0}}$ at depth *z*_0_ < *z* which corresponds to the reference level for export (e.g., bottom of the epipelagic zone). It reads as

(1) $$F\left(z\right)=F_{z_{0}}{\left(\frac{z}{z_{0}}\right)}^{-b}$$

where *b* is the flux attenuation exponent (often referred to as the “*Martin* *b* *parameter*”), which depends on the balance between POC sinking speed and rate of remineralization (Middelburg, 2019). In fact, ignoring the effects of circulation on POC, if we assume a sinking velocity that increases linearly with depth of the form *w*(*z*) = *az*, with *a* > 0 constant, and a constant decay rate *λ* > 0, then Equation 1 is obtained as an exact annually averaged steady state solution for an advection‐reaction equation, with *b* = *λ*/*a* (see also Equation 3 in Methods). Note that using Equation 1,

(2) $$\text{TE}=\frac{F\left(z=1,000m\right)}{F_{z_{0}}}={\left(\frac{z=1,000m}{z_{0}}\right)}^{-b}.$$

In Martin's original work, for the parameter *b* the limited observations in the Pacific Ocean suggested a value of 0.858 as the best fit—with the caveat that the trap data from coastal sites reported in the same study were not used to calculate the exponent and did not fit this value. Since then, this parameterization has been widely adopted and is an integral part of many global biogeochemical models, particularly those that do not model detritus explicitly (Kriest & Oschlies, 2008). For those models that do represent detritus explicitly, this constant Martin *b* = 0.858 is usually used to specify the sinking speed rate *a* through the aforementioned equation *b* = *λ*/*a* (see for instance Kriest & Oschlies, 2015) as this model‐imposed *b* equals the diagnosed *b* by Equation 1 for a model with *a* = *λ*/*b* in the absence of circulation effects (see also Section 2.1).

In this work, our approach consists of adding seasonal variability to the flux attenuation parameter *b*—here denoted by *b* ^model^ to distinguish from the *b* that is diagnosed from Equation 1—in the numerical ocean‐biogeochemical model described in Section 2.1. This seasonality (see Section 2.2) is varied in both strength and phase relative to solar radiation, and the resulting effects are compared to those observed in the case of a constant *b* ^model^ as explained in Section 2.3. The metrics used and their underlying assumptions are presented in Section 2.4. All spatial plots shown in this manuscript uses the colormap package developed by Thyng et al. (2016).

### 2.1 Model

This section provides a brief description of the modeling framework used. In order to run several experiments covering a wide range of parameter values, we use the Transport‐Matrix Method (TMM; Khatiwala, 2007; Khatiwala et al., 2005). This is a fast, efficient, seasonally varying, offline representation of the ocean circulation derived from a global circulation model, which is freely available online (see Khatiwala, 2018). Here, 12 monthly averaged transport matrices computed by Khatiwala et al. (2005) from the MIT Global Circulation Model are used to recreate the annual circulation. The configuration used is global, has 15 vertical layers and 2.8° spatial resolution. For more on the TMM, the reader is encouraged to see Khatiwala et al. (2005), and Khatiwala (2007); Khatiwala (2018).

For the biogeochemistry, we use the GEOMAR NPZD‐DOP Biogeochemical Model (Kriest & Oschlies, 2011; Kriest et al., 2010, 2012). This model is based on the production, export and remineralization of organic phosphorus, with phosphate (PO_4_, given in in mmol P m^−3^) as a nutrient, which for this study is converted into carbon using the Redfield ratio C:N:P = 106:16:1. In this model, the remineralization of organic matter is represented by the decay of detritus (in mmol P m^−3^), which is treated explicitly as a tracer (see Equation 27 in Kriest et al., 2010). This detritus is generated by dying and eaten phytoplankton and sinks with speed *w* (in day^−1^ m^−1^)—which in turn is assumed to increase linearly with depth such that *w* = *az* (*a* > 0)—while decaying at specific remineralization rate *λ* (taken to be 0.05 day^−1^).

The crucial point here is that the explicit treatment of the detritus dynamics effectively imposes a power law curve on the flux profile, which in the absence of circulation, converges to the annually averaged steady state profile given by Equation 1 with *b* = *λ*/*a* as before. This deterministic relation allows one to specify a priori a value to *b*, and in this way impose time variability on the sinking speed via

(3) $$a=\frac{\lambda }{b}.$$

Although Equation 3 has been widely used in models that represents detritus explicitly (for example Kriest & Oschlies, 2015), this relation between the constant exponent *b* in Equation 1 and the model‐imposed constant sinking speed rate *a* is only true in the absence of circulation. When circulation is present, the value of *b* imposed by Equation 3 and the value of *b* that is inferred via Equation 1 do not match. For this reason, we shall rename the parameter *b* present in the model as *b* ^model^.

In this model, *a* is determined by the already specified *λ* = 0.05 day^−1^ and a reference value for *b* ^model^ given by $b^{\text{model}}=b_{ref}^{\text{model}}=1.388$ (both values coming from Kriest, 2017). This gives a value of approximately 0.036 day^−1^ for *a*. When compared to the World Ocean Atlas 2018 climatology (Garcia et al., 2018), a value of $b_{ref}^{\text{model}}=1.388$ gives a better agreement to PO_4_ distributions than the Martin value of 0.858 (Martin et al., 1987) as indicated in Figure 2 we should note that this model is phosphate‐based, while the Martin et al. (1987) *b* = 0.858 value was obtained for carbon, and that there is evidence that the attenuation profiles for both might differ in certain regions of the ocean (see e.g., Figure 2 of Engel et al., 2017). The fidelity of the calibrated model to observations is not so relevant to this study, since the model is being used to explore behavior rather than to precisely match observations. A complete description of the NPZD‐DOP model can be found at Kriest et al. (2010); Kriest et al. (2012), Kriest and Oschlies (2011) and includes details on how the computation of the depth *z* for the sinking speed is numerically handled at each vertical grid box to prevent overestimation of fluxes (Kriest & Oschlies, 2011).

Finally, it is important to note again that if modeled fluxes are used to diagnose *b* via Equation 1, and consequently TE, then the results are emerging properties of the coupled ocean‐biogeochemical model and cannot be determined by the imposed form of *b* ^model^ solely. Therefore, the “emergent” *b* from Equation 1 cannot be imposed, since the POC dynamics is influenced by the background ocean circulation. Equation 3 summarizes only the contribution of the biogeochemical module to the POC sinking under the hypothesis of constant *a*, *λ* and *b* and does not account for the physical circulation. In other words, *b* ^model^ ≠ *b* in general. This fact is largely neglected in current literature and we discuss its consequences in Sections 3 and 4.

### 2.2 Parameterizations

At the core of this study we wish to apply a seasonal (periodic) perturbation to flux attenuation and we do this by applying to *b* ^model^ (denominator of Equation 3) a perturbation (or “seasonal variation”) of magnitude $\tilde{b}\left(t\right)$, such that the sinking speed *w*(*z*) = *az* is replaced by a time‐dependent sinking speed *W*(*z*, *t*) = *A*(*t*)*z*, with

(4) $$A\left(t\right)=\frac{\lambda }{b^{\text{model}}+\tilde{b}\left(t\right)}=\frac{\lambda }{b^{\text{model}}}+\tilde{a}\left(t\right)=a+\tilde{a}\left(t\right).$$

Equation 4 above shows that adding a seasonal variation $\tilde{b}\left(t\right)$ to the constant flux attenuation coefficient *b* ^model^ is equivalent to adding a seasonal variation $\tilde{a}\left(t\right)$ to the sinking speed coefficient *a*. Equation 4 is consistent with the hypothesis of a seasonally varying sinking speed that increases with depth, which has been recently confirmed in a North Atlantic‐based work by Villa‐Alfageme et al. (2016). The assumption of a constant *λ* is addressed in the discussion in Section 4.5.

**Figure 1:**
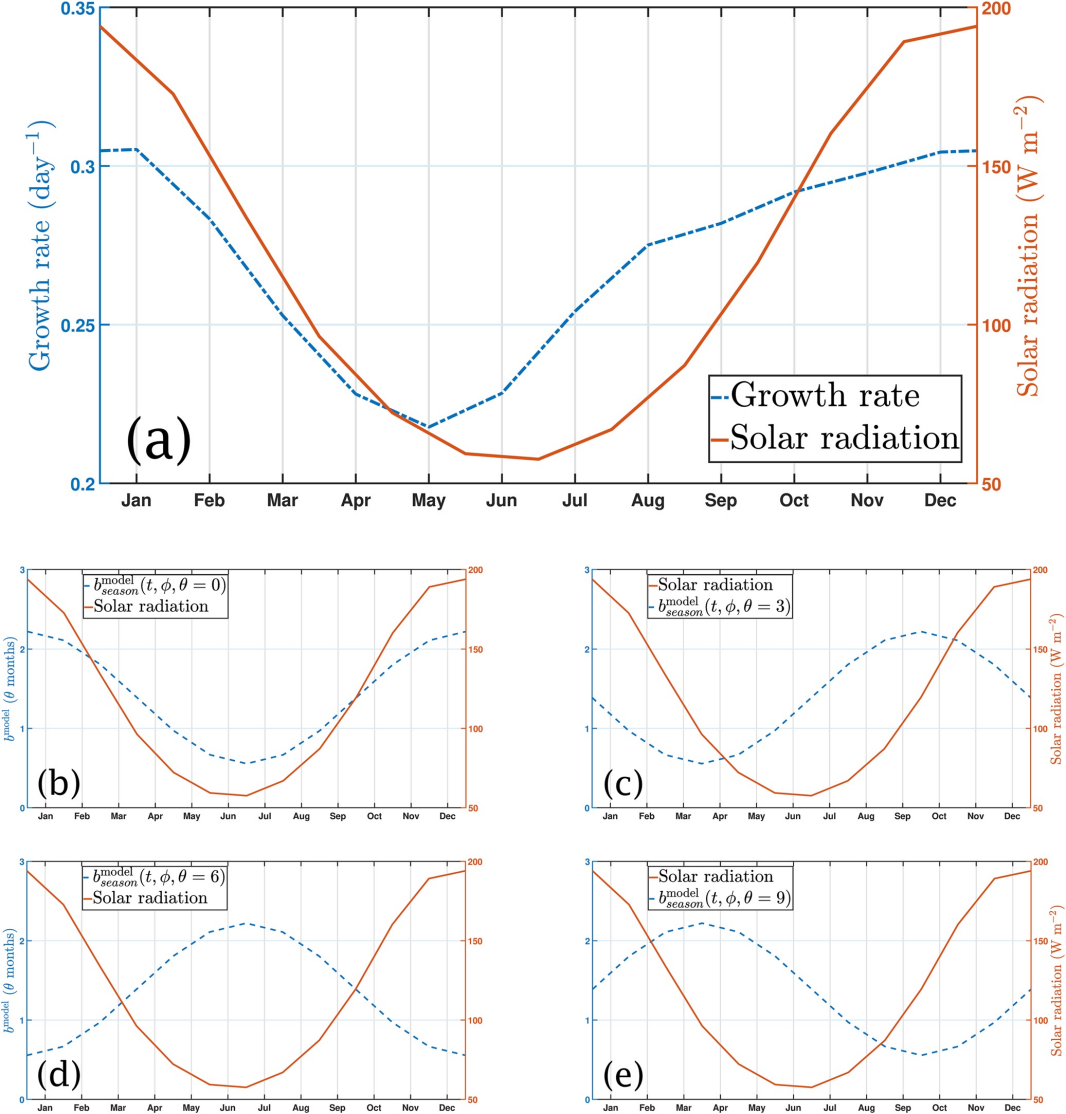
Top figure shows: (a) Schematic comparison between the daily average shortwave solar radiation at the sea surface (W m^−2^) and phytoplankton growth (day^−1^) in the Southern Hemisphere, for *b* ^model^ = 1.388. Bottom shows a comparison of averaged solar radiation and $b_{season}^{model}$ in the Southern Hemisphere (*ϕ* < 0) during a year, for different phases *θ* (in months): (b) *θ* = 0, $b_{season}^{\text{model}}$ and solar radiation in phase; (c) *θ* = 3, maximum $b_{season}^{model}$ after summer; (d) *θ* = 6, opposite phases; and (e) *θ* = 9, maximum $b_{season}^{model}$ before summer.

In order to compare the effects of seasonality to the case of constant, uniform flux attenuation, we test a modified version of *b* ^model^. This new parameterization applies a cosine oscillation to the *b* ^model^ parameter. Specifically, we take

(5) $$b_{season}^{\text{model}}\left(t,\phi ,\theta \right)=b^{\text{model}}+\tilde{b}\left(t\right)=\left\{\begin{array}{cc} b_{ref}^{\text{model}}-\delta bcos\left(2\pi \left(t/T\right)+\left(\theta \pi /6\right)\right),\; & \text{if}\;\phi >0\\ b_{ref}^{\text{model}}+\delta bcos\left(2\pi \left(t/T\right)+\left(\theta \pi /6\right)\right),\; & \text{if}\;\phi <0\\ b_{ref}^{\text{model}},\; & \text{if}\;\phi =0 \end{array}\right.$$

where *t* is the time (in days), *T* is the period of one seasonal cycle (in days), *ϕ* is the latitude in degrees North, $b_{ref}^{\text{model}}>0$ is a reference (mean) value for *b* ^model^, $\tilde{b}\left(t\right)$ takes the time‐varying form shown, $\delta b\leq b_{ref}^{\text{model}}$ is the magnitude of the seasonal effect and *θ* is the phase, defined here as the time in months of the minimum in $b_{season}^{\text{model}}$ in the Northern Hemisphere relative to 1 January. A comparison of the different phases with respect to solar radiation in the Southern Hemisphere is shown in Figure 1. Note that, in this model, 1 year corresponds to 360 days, and hence *T* = 360 days.

**Figure 2:**
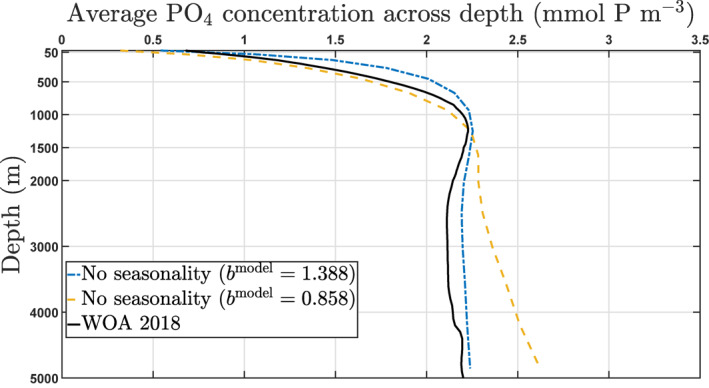
Global annual average PO_4_ concentration (mmol P m^−3^) versus depth. Black solid line shows the World Ocean Atlas 2018 climatology. The yellow dash and blue dash‐dot lines show the model average concentration for a constant *b* ^model^ equal to 0.858 and 1.388, respectively.

From the above, it is important to note that, while the annual mean value of $b_{season}^{\text{model}}\left(t,\phi ,\theta \right)$ given by Equation 5 is (for any choice of *ϕ* and *θ*) equal to $b_{ref}^{\text{model}}$, the mean value of *A*(*t*) in Equation 4, which we will denote by $\bar{A}$, does not equal $\lambda /b_{ref}^{\text{model}}$. This means that a periodic and symmetrically varying *b* ^model^ gives a periodic but asymmetric sinking speed coefficient *A*(*t*). For instance, for $b_{ref}^{\text{model}}=1.388$, we have that

$$\bar{A\left(t\right)}=\left(\frac{1}{T}\right)\int_{0}^{T}A\left(t\right)dt=\left(\frac{1}{T}\right)\int_{0}^{T}\left(\frac{\lambda }{b_{season}^{\text{model}}\left(t,\phi ,\theta \right)}\right)dt\approx 0.045{day}^{-1},$$

for any *ϕ* ≠ 0 and *θ*, which gives a sinking speed that is 20% faster than the one given by taking *a* = 0.036 day^−1^ as used by the model in the non‐seasonal case. The corresponding value of *b* ^model^ for this value of $\bar{A}$ is

$$b^{\text{model}}=\frac{\lambda }{\bar{A\left(t\right)}}=\frac{0.05}{0.045}\approx 1.110,$$

which gives a flux profile where remineralization occurs deeper than for *b* ^model^ = 1.388, consistent with it sinking faster. In other words, the introduction of a cosine varying seasonality in *b* ^model^ increases the annual mean POC flux at any depth below 120 m (see, for instance, Figures 2 and 5).

We therefore also examine the case where *A*(*t*) varies seasonally about $a=\lambda /b_{ref}^{\text{model}}=0.036\;{day}^{-1}$ such that the annual average of *A*(*t*) is $\bar{A\left(t\right)}=0.045\;{day}^{-1}$. In this case the annual average of *b* ^model^ is not 1.388 but 1.110 as described above.

For simplicity, from now on we will omit the independent variables *t*, *ϕ* and *θ* when referring to the parameterization above (e.g., $b_{season}^{\text{model}}$ instead of $b_{season}^{\text{model}}\left(t,\phi ,\theta \right)$).

### 2.3 Experiments

The parameterization given by Equation 5 has three important parameters: the reference flux attenuation $b_{ref}^{\text{model}}$, the seasonal phase *θ* and the seasonality strength *δb*. While $b_{ref}^{\text{model}}$ is fixed, the latter two parameters control the seasonal variability in *b* ^model^. In order to assess the influence of each of these parameters, we run the following series of experiments:

1. Seasonal phase *θ* (phasing of phytoplankton growth relative to time of year). We test 4 different seasonal phases with respect to the 1 January, as shown in Figure 1. These are: *θ* = 0 months (shown in Figure 1b), which in the Southern hemisphere corresponds to a maximum (minimum) flux attenuation *b* ^model^ on 1 January (1 July); *θ* = 3 months (see Figure 1c), where a maximum (minimum) *b* ^model^ occurs at the start of October (April); *θ* = 6 months, where maximum solar irradiation matches minimum *b* ^model^ and vice‐versa (Figure 1d); and *θ* = 9 months where the Southern hemisphere summer solstice radiation happens 3 months after the maximum *b* ^model^ (Figure 1e).
2. Seasonal strength *δb* (strength of seasonal variability in *b* ^model^). We focus on simulating the case of $\delta b=0.6b_{ref}^{\text{model}}$ (60% of variation at peak values). For $b_{ref}^{\text{model}}=1.388$, this value of *δb* corresponds to *b* ^model^ varying from 0.555 to 2.221, which roughly matches the range reported of 0.5–2.0 reported in the Introduction. To assess the sensitivity of variations in *δb*, we also simulate the cases of $\delta b=0.2b_{ref}^{\text{model}}$ (*b* ^model^ varying from 1.110 to 1.666) and $\delta b=0.4b_{ref}^{\text{model}}$ (*b* ^model^ varying from 0.833 to 1.943), which are presented in the Supporting Information S1.
3. Non‐seasonal runs. As a reference to compare to the seasonal runs described above, we perform non‐seasonal experiments (i.e., *δb* = 0) for selected values of $b_{ref}^{\text{model}}$. These are: $b_{ref}^{\text{model}}=1.388$, which is the reference value for *b* ^model^; $b_{ref}^{\text{model}}=0.555$ and $b_{ref}^{\text{model}}=2.221$, which are respectively the minimum and maximum values assumed by $b_{season}^{\text{model}}$ when seasonality in *b* ^model^ is 60% of the reference value; and $b_{ref}^{\text{model}}=1.110$, which is the value of *b* ^model^ such that *λ*/*b* ^model^ gives the 1‐year mean value of *A*(*t*) as discussed previously (see Section 2.2)

In terms of the sinking speed rate *A*(*t*), a seasonal strength of $\delta b=0.6b_{ref}^{\text{model}}$ translates to *A*(*t*) ranging between a minimum of approximately 0.023 day^−1^ (when *b* ^model^ = 2.221) to a maximum of approximately 0.090 day^−1^ (when *b* ^model^ = 0.555). This means that, over a 1‐year period, the seasonally varying sinking speed *W*(*t*) = *A*(*t*)*z* at 120 m varies between 2.70 and 10.81 m day^−1^, and this range increases linearly to 11.26 and 45.05 m day^−1^ at the mesopelagic depth of 500 m, and to 24.31 and 97.30 m day^−1^ at 1,080 m. These values are consistent with what has been reported in literature (Villa‐Alfageme et al., 2016), with sinking speeds of less than 10 m day^−1^ observed in locations such as the North Atlantic (Riley et al., 2012) and the subtropical Atlantic (Alonso‐González et al., 2010), and from 25 to 150 m day^−1^ in the Southern Ocean (McDonnell & Buesseler, 2010), and also with what has been used in models ‐ for instance, the UKESM‐adopted biogeochemical model MEDUSA uses a fixed value of 3 m day^−1^ as the sinking speed for its slow pool of detritus (Henson et al., 2015; Yool et al., 2013).

Similarly, for the sensitivity experiments, when $\delta b=0.2b_{ref}^{\text{model}}$ we have *A*(*t*) ranging between 0.030 day^−1^ (when *b* ^model^ = 1.666) to 0.045 day^−1^ (when *b* ^model^ = 1.110); and for $\delta b=0.4b_{ref}^{\text{model}}$ we have *A*(*t*) ranging between 0.026 day^−1^ (when *b* ^model^ = 1.943) to 0.060 day^−1^ (when *b* ^model^ = 0.833).

Each experiment is run for 3,000 years to reach a consistently quasi‐repeating annual cycle, as per Kriest et al. (2010).

### 2.4 Metrics and Assumptions

The effects of seasonality are measured through the following quantities (here, $\bar{F}$ and $\bar{\text{PP}}$ denote the annual averages of both POC flux and primary production, respectively, at any location [*x*, *y*, *z*]): the total net primary production PP_global_ is given by

(6) $${\text{PP}}_{\text{global}}=\int \bar{\text{PP}}\left(x,y,z\right)dxdydz;$$

the local transfer efficiency TE is defined as

(7) $$\text{TE}\left(x,y\right)=\frac{\bar{F}\left(x,y,z=1,080m\right)}{\bar{F}\left(x,y,z=120m\right)};$$

the global fluxes at 120 m and 1,080 m are respectively given by

(8) $$F_{120m}=\int \bar{F}\left(x,y,z=120m\right)dxdy\;\text{and}\;F_{1,\;080m}=\int \bar{F}\left(x,y,z=1080m\right)dxdy;$$

and the global transfer efficiency TE_global_ is given by

(9) $${\text{TE}}_{\text{global}}=\frac{F_{1080m}}{F_{120m}},$$

where the values of *z* = 120 m for export depth and *z* = 1,080 m for transfer depth are imposed by the depth of model layers, as these are the depths where the diagnostic fluxes are computed.

We note that, while sinking to depth, organic material such as POC is also advected horizontally and therefore the material initially at a location (*x*_0_, *y*_0_) at *z*_0_ = 120 m might no longer be at (*x*_0_, *y*_0_) when it reaches *z* = 1,080 m. We discuss the consequences of this in Section 4.

Finally, we only use output where the model is at least 1,080 m deep. This excludes shallow areas such as shelves and coastal locations, but including them would introduce a significant numerical bias to the PP and export flux relative to the flux at 1,080 m.

## 3 Results

In this section, we present and describe the results of the numerical simulations performed to assess the influence of seasonal variations in *b* ^model^ and sinking speed.

### 3.1 Global and Regional PO_4_ Distributions

**Figure 3:**
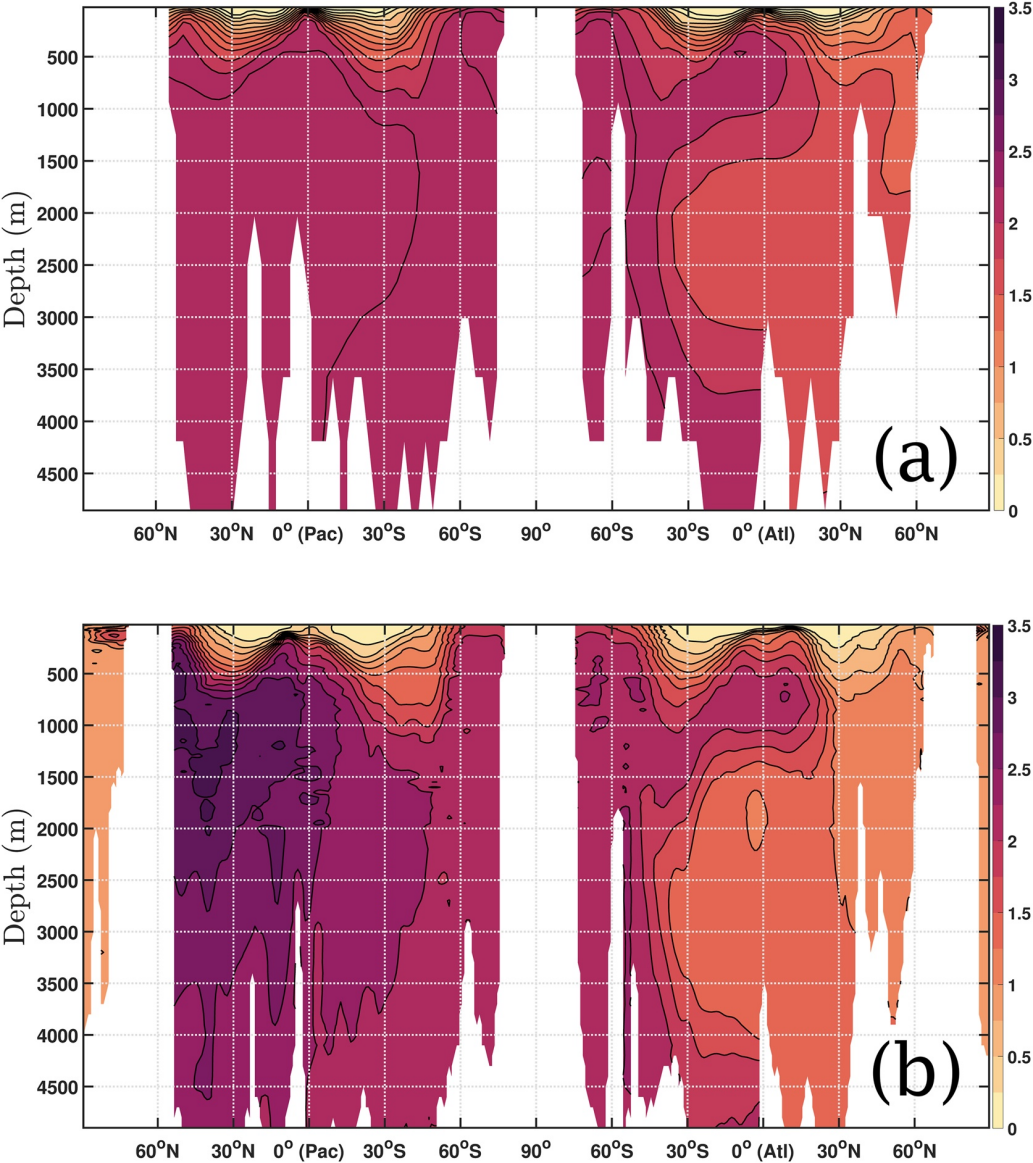
Cross‐section of the global annual average PO_4_ concentrations field (mmol P m^−3^) for both: (a) the case of constant $b^{\text{model}}=b_{ref}^{\text{model}}=1.388$; and (b) World Ocean Atlas 2018 climatology. The Pacific ocean section is taken at longitude 159°W (shown on the left‐hand side) and the Atlantic Ocean is cross‐sectioned at longitude 25°W (on the right‐hand side).

The distribution of phosphate for the non‐seasonal case of *b* ^model^ = 1.388 is presented in Figure 3a, which shows a transect of the Atlantic Ocean along longitude 25°W connecting to a transect across the Pacific Ocean at 159°W. Overall, it shows a good agreement with the spatial pattern in the WOA climatology (Garcia et al., 2018) shown in Figure 3b, except for the North Pacific where the model underestimates PO_4_ concentrations.

**Figure 4:**
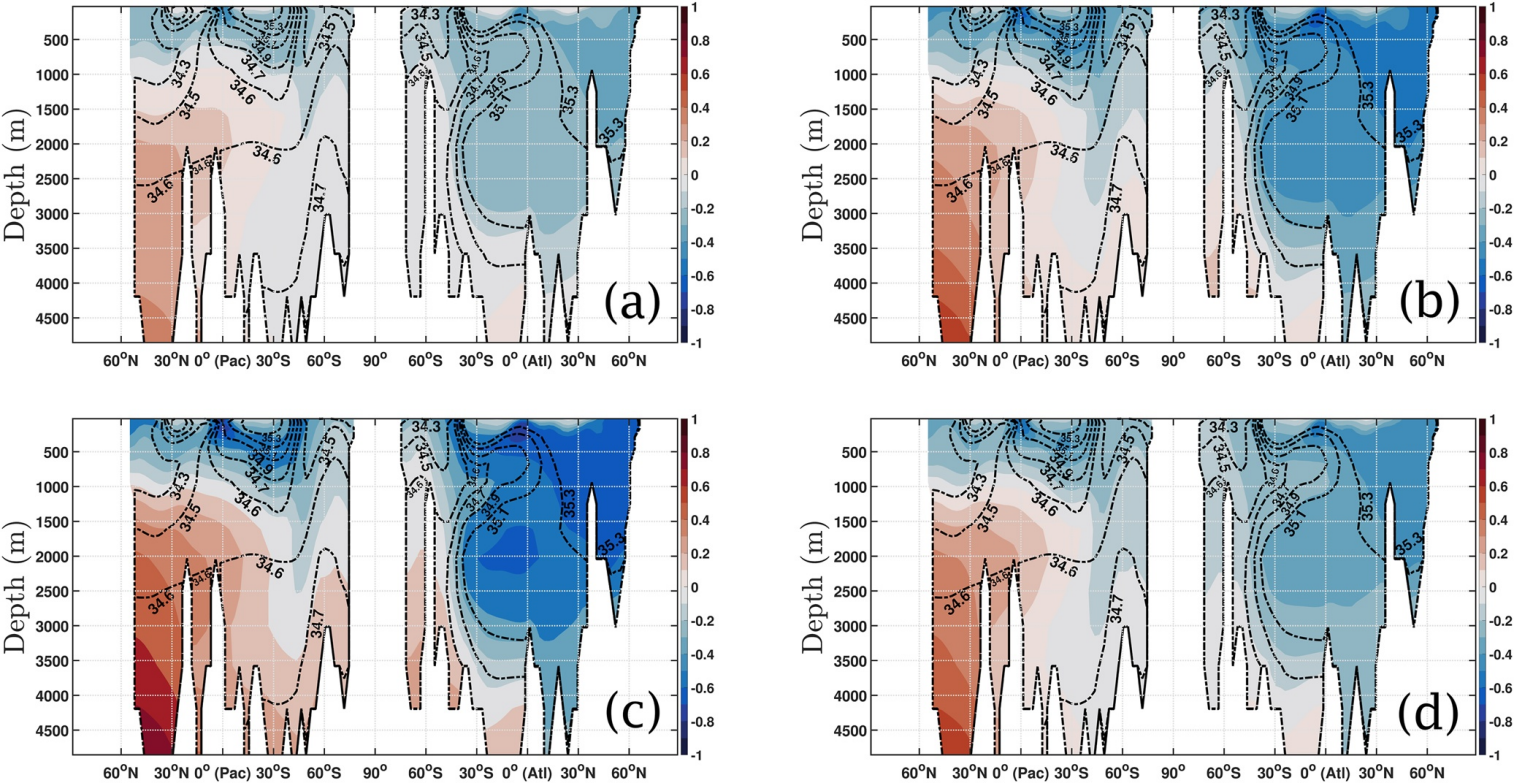
Changes in PO_4_ concentration (mmol P m^−3^) for a cross‐section of the global ocean (in each figure, the Pacific is on the left and the Atlantic is on the right) for $b_{season}^{\text{model}}$ with a seasonality of 60% and several values of *θ* (in months), when compared to the non‐seasonal case shown in Figure 3a: (a) *θ* = 0 months; (b) *θ* = 3 months; (c) *θ* = 6 months; and (d) *θ* = 9 months. All figures have salinity field contour lines (dash‐dot black lines) superimposed. These figures are also reproduced as Figures S7–S10 in Supporting Information S1, with additional plots showing the density fields superimposed.

The introduction of a global seasonal perturbation in *b* ^model^ changes substantially the PO_4_ concentrations with respect to the constant *b* ^model^ simulation. Figure 4 shows the changes in concentration with respect to the non‐seasonal case of Figure 3a, when allowing the seasonality to change the base value of *b* ^model^ = 1.388 by 60% $\left(\delta b=0.6b_{ref}^{\text{model}}\right)$. Generally, concentrations are increased deeper than 2,000 m in the Pacific, and decreased in the Atlantic down to 4,000 m. This pattern holds for all *θ*, with the greatest magnitude of changes for *θ* = 6 months (Figure 4c), which corresponds to maximum *b* ^model^ occurring on 1 January (1 July) in Northern (Southern) Hemisphere. In the latter, an increase of over 0.8 mmol P m^−3^ is observed in the very deep waters of the temperate North Pacific, with a 0.6 mmol P m^−3^ decrease seen across the mesopelagic Atlantic Ocean section. A high variability is also observed for *θ* = 3 months and *θ* = 9 months, as shown in Figures 4b and 4d respectively. A smaller change is observed when *θ* = 0 months (Figure 4a), where the variability is constrained to a maximum of 0.3 mmol P m^−3^ in most of the transect. Note that, whatever the value of *θ*, the presence of a global seasonality increases the concentration in the very deep waters (below 2,000 m) of the North Pacific by at least 0.4 mmol P m^−3^ and almost doubles it for certain values of *θ*. It also decreases consistently the concentrations in most of the mesopelagic and deep Atlantic, excluding the Southern Ocean.

**Figure 5:**
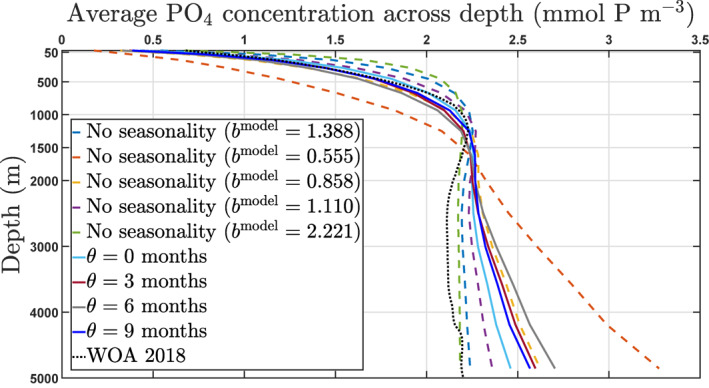
Annual global average PO_4_ concentration (mmol P m^−3^) at each depth in the global ocean. Solid lines show the concentration profile for each phase *θ*. The blue and purple dash lines show the average concentration when a constant *b* ^model^ equals 1.388 and 1.110, respectively. The red and green dash lines show the concentration for a constant *b* ^model^ equal to the minimum (0.555) and maximum (2.221) of $b_{season}^{\text{model}}$ across the year, respectively. The yellow dash lines show the concentration for a constant *b* ^model^ equal to the Martin value of 0.858. The dotted black line shows the concentration profile obtained from the WOA 2018.

The global average concentration of PO_4_ at each depth is also significantly affected by the introduction of seasonality. Consistent with the structure seen in Figure 4, Figure 5 shows that the presence of seasonality tends to decrease the global average PO_4_ concentration in the upper 1,500–2,000 m but increases it substantially below 2,000 m depth. These seasonal profiles shown in Figure 5 (solid lines) fall in between the profiles given by scenarios of constant *b* ^model^ = 0.555 (dash‐red line) and *b* ^model^ = 2.221 (dash‐green line), which respectively correspond to the minimum (*b* ^model^ = 1.388 minus 60%) and maximum (*b* ^model^ = 1.388 plus 60%) values attained by the seasonal *b* ^model^ (with seasonal variability of 60% of $b_{ref}^{\text{model}}$) across the year.

### 3.2 Influence of Seasonality on TE

**Figure 6:**
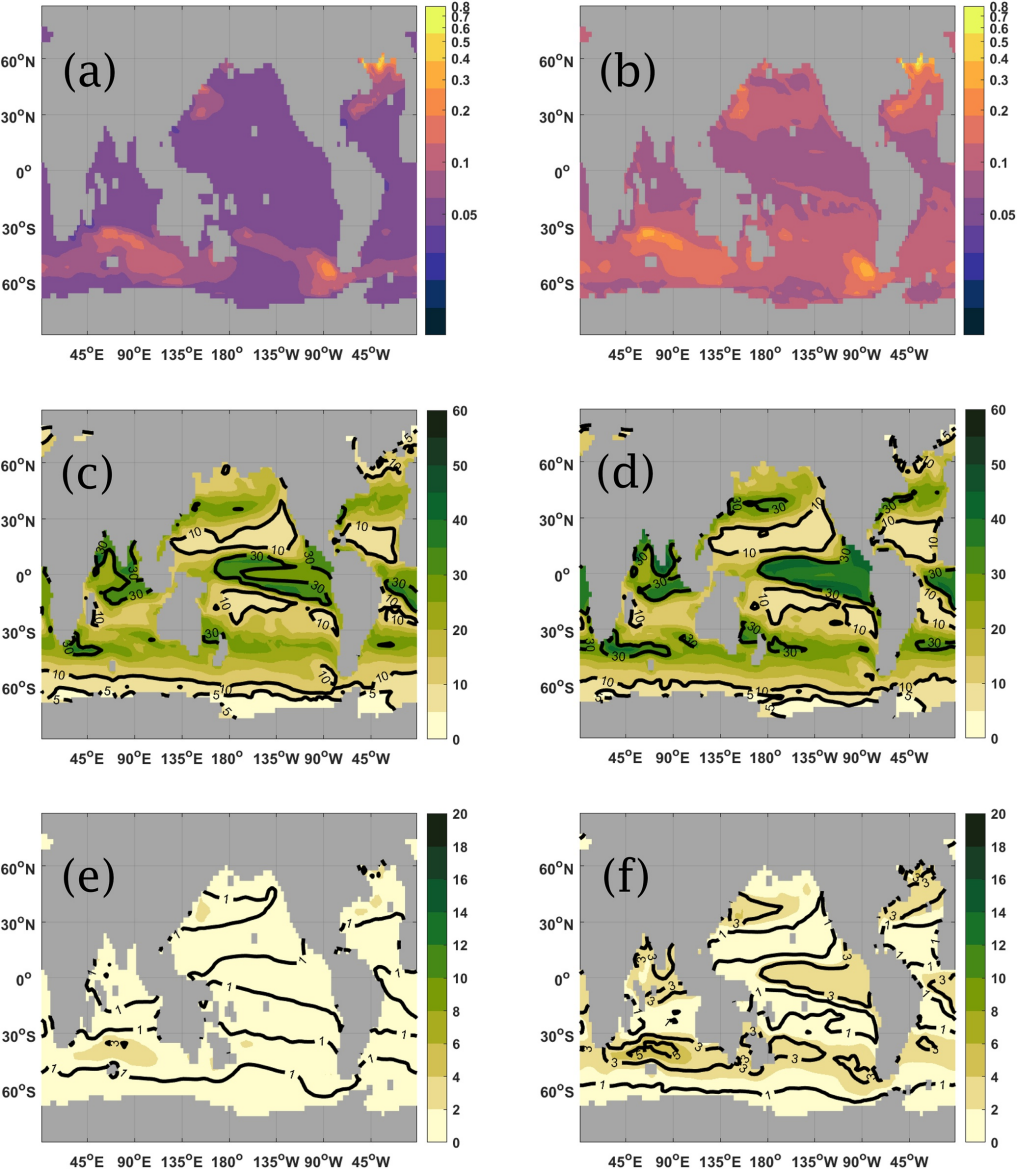
1‐year average transfer efficiency (TE) (Figures 6a and 6b) for the case of constant *b* ^model^ and 1‐year average fluxes (g C m^−2^ year^−1^) at both 120 m (Figures 6c and 6d) and 1,080 m (Figures 6e and 6f) depth. The column on the left‐hand side show both TE and fluxes for *b* ^model^ = 1.388, while the right‐hand side show the corresponding images for *b* ^model^ = 1.110. Note the different color bar limits in plots 6c to 6f.

The global maps for TE and fluxes in the non‐seasonal cases of *b* ^model^ = 1.388 and *b* ^model^ = 1.110 are presented in Figure 6. Figures 6a and 6b show the global map of TE for *b* ^model^ = 1.388 and *b* ^model^ = 1.110, obtained from Equation 7, which present a spatial pattern despite the applied *b* ^model^ being uniform. We first note that, when *b* ^model^ = 1.388 the global mean for this diagnosed TE is 0.066, which is over 40% higher than the 0.047 predicted by the Martin curve (as per Equation 2), which ignores the effect of circulation (see Discussion). Similarly, for *b* ^model^ = 1.110 the diagnosed TE from the figure is 0.11, which is 25% higher than the Martin curve prediction of 0.088. Looking into these regional patterns, we note that for *b* ^model^ = 1.388 this model‐diagnosed TE remains at around 0.05–0.06 in most of the tropical and subtropical ocean, as well as in parts of the North Pacific and in narrow areas of the Southern Ocean near the Antarctic coast. In the temperate and polar areas, particularly in the Southern Ocean, the TE map shows a value of between 0.1 and 0.2, except for a few localized hotspots located in the North Atlantic (60°N), Northwest Pacific (30°N), Southern Indian (40°S) and Southeast Pacific (55°S), where high TE values of 0.3–0.4 and above are seen. A similar spatial pattern is observed when *b* ^model^ = 1.110.

When looking separately at the POC fluxes at both 120 m and 1,080 m, presented for *b* ^model^ = 1.388 in Figures 6c and 6e, we observe that they present a similar pattern. However, we also note differences in spatial gradients at the two depths. For instance, the flux at 120 m in the Pacific Southern Ocean area (between 180°W and 90°W) shows a gradient from 5 to 10 g C m^−2^ year^−1^ (an increase of 100%) while the flux at 1,080 m shows a variability from 0.2 to 1 g C m^−2^ year^−1^ in the same area which results in a higher transfer as per Equation 7. More generally, the fluxes are higher in both tropical and temperate regions and are low in the nutrient‐poor subtropical gyres as expected. However, TE does not show this same pattern, being around 0.055 throughout the ocean, and therefore spatial patterns in productivity at the surface do not necessarily relate to those in TE. The fluxes for *b* ^model^ = 1.110 shown in Figures 6d and 6f present a similar behavior.

**Figure 7:**
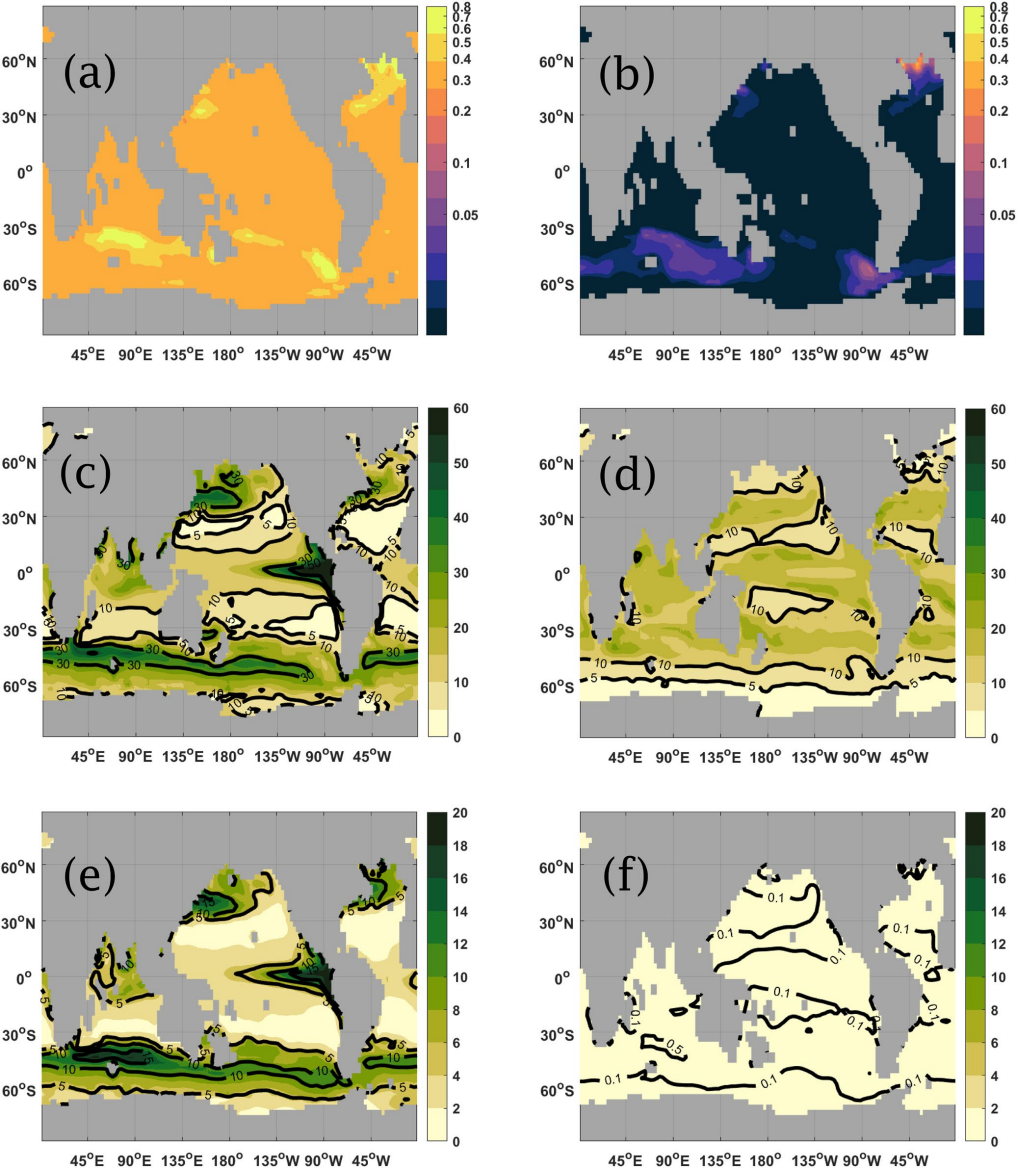
1‐year average transfer efficiency (TE) (Figures 7a and 7b) for the case of constant *b* ^model^ and 1‐year average fluxes (g C m^−2^ year^−1^) at both 120 m (Figures 7c and 7d) and 1,080 m (Figures 7e and 7f) depth. The column on the left‐hand side show both TE and fluxes for *b* ^model^ = 0.555, while the right‐hand side show the corresponding images for *b* ^model^ = 2.221. Note the different color bar limits in plots 7c to 7f.

A similar qualitative behavior (in TE and fluxes at both 120 m and 1,080 m depth) is observed in the non‐seasonal cases of $b^{\text{model}}=b_{ref}^{\text{model}}=0.555$ and $b^{\text{model}}=b_{ref}^{\text{model}}=2.221$, which correspond to the extrema of the seasonal varying *b* ^model^ with $\delta b=0.6b_{ref}^{\text{model}}$. These are shown respectively on the left and right columns of Figure 7. We note that for *b* ^model^ = 0.555, which implies a faster sinking speed globally (as per Equation 3), the overall TE of around 0.33 is observed, and the regional patterns in TE are weaker, giving a more homogeneous global map for TE. On the other hand, for *b* ^model^ = 2.221, which corresponds to a very slow sinking speed (as per Equation 3), we obtain a value of TE below 0.010 in many areas but now with a more pronounced regional variability in TE, including in the subtropical areas which present near‐zero transfer efficiency. This highlights the effects of the circulation moving material around as it sinks, which is returned to in the Discussion. The globally averaged TE for *b* ^model^ = 0.555 from Figure 7a is 0.35, which is closer to Equation 2 estimate of 0.29. For *b* ^model^ = 2.221 from Figure 7b, the globally averaged TE is 0.016, which is 110% higher than the 0.0076 given by Equation 2.

**Figure 8:**
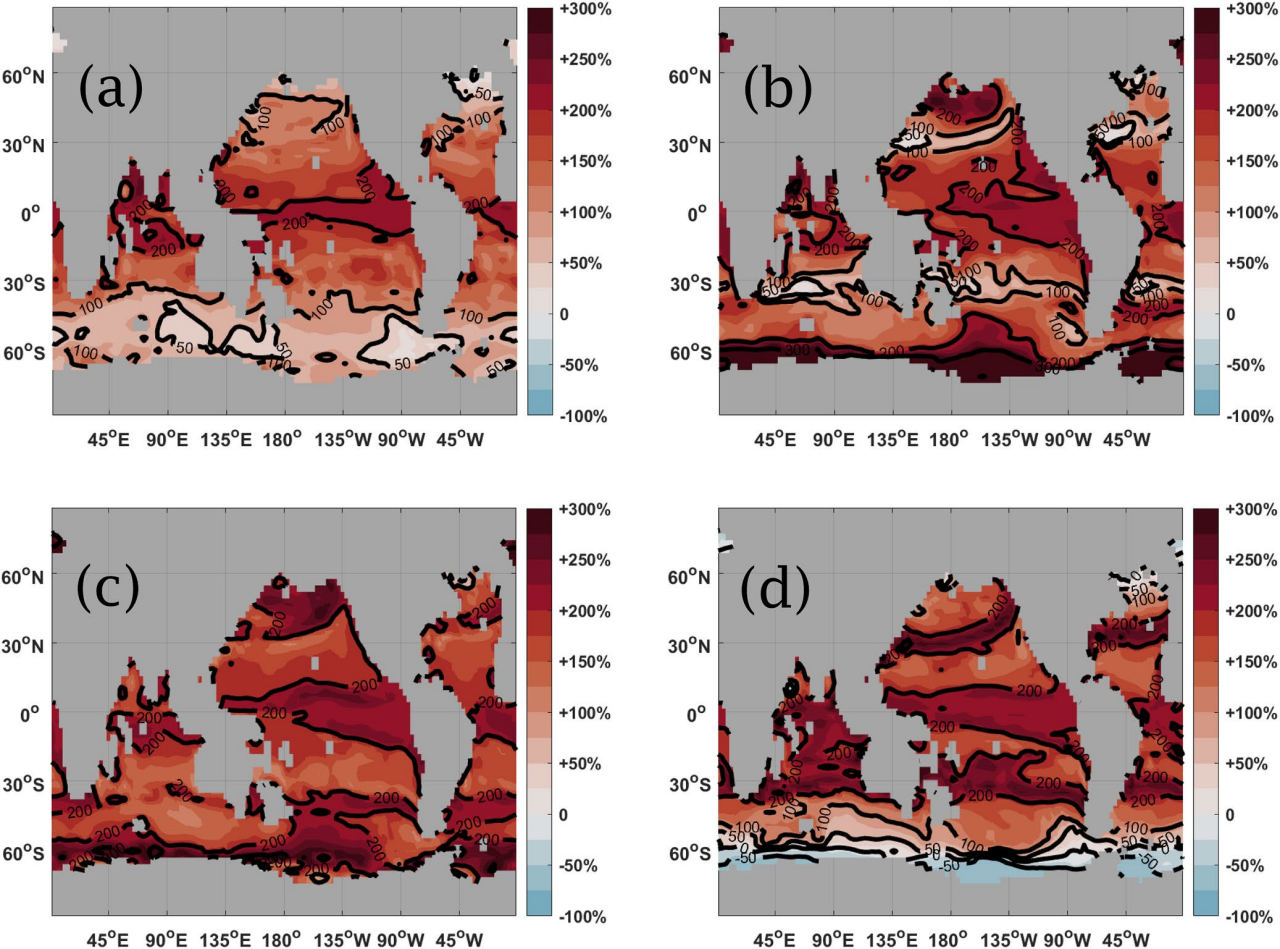
Percentage change (when compared to the non‐seasonal case) in annual transfer efficiency (TE) for the parameterization $b_{season}^{\text{model}}$ with $\delta b=0.6b_{ref}^{\text{model}}$, when compared to the non‐seasonal case. Figure 8a: *θ* = 0 months; Figure 8b: *θ* = 3 months; Figure 8c: *θ* = 6 months; Figure 8d: *θ* = 9 months; The solid black contour curves highlight areas of zero increase (e.g., Southern Ocean in [d]) and of 200% increase (e.g., Equator in [a]). Some of the dark red areas of high‐TE have a TE increase in excess of 300% in some singular locations near the coast.

The influence of seasonality on TE is presented in Figure 8 as the fractional change in TE relative to the non‐seasonal case of *b* ^model^ = 1.388 shown in Figure 6a. The same comparison is made with respect to the the non‐seasonal case of *b* ^model^ = 1.110 shown in Figure 6b and is available in the Supporting Information S1. Broadly, Figure 8 shows an increase in TE at both low and high latitude areas, with the largest effects observed when *θ* = 6 months (corresponding to a maximum *b* ^model^ in July in the Southern Hemisphere and January in the Northern Hemisphere), and the smallest seen when *θ* = 0 months (maximum *b* ^model^ in January in the Southern Hemisphere and July in the Northern Hemisphere) and *b* ^model^ is nearly in phase with primary production (as per Figure 1a).

Across the different cases in Figure 8, the Southern Ocean shows a rather distinctive behavior. There is a consistent increase of over 200% (over 300% in some areas) when maximum *b* ^model^ occurs in either winter (*θ* = 6 months) or spring (*θ* = 3 months), and also a widespread decrease of more than 50% for a *b* ^model^ peaking in autumn (*θ* = 9 months), with a more homogeneous variability of 50%–100% seen when *b* ^model^ reaches its maximum in January (*θ* = 0 months).

In terms of global impact, Tables 1 and 2 show the values of TE_global_ for non‐seasonal and seasonal cases respectively. When seasonality is not present, a decrease in *b* ^model^ by 60% (*b* ^model^ = 0.555) increases the transfer efficiency by a disproportional 478%, while an increase in *b* ^model^ by the same percentage (*b* ^model^ = 2.221) decreases TE by nearly 80%. This is expected because of the nonlinear influence that *b* ^model^ has on the sinking speed as per Equation 3, in which small values of *b* ^model^ (which has a minimum of zero) increase the sinking speed without limit, while high values of *b* ^model^ (which can be as high as one wishes) decrease the sinking speed down to zero at most. For the seasonal cases, on the other hand, the general trend is of a consistently higher TE. Just the presence of seasonality increases the global transfer efficiency by at least 145%, regardless of the relative phase *θ*, with nearly a two‐fold boost observed when *θ* = 6 months. This is in part due to the fact that the average value for the seasonally varying sinking speed (denoted by $\bar{A\left(t\right)}$) is 20% higher than the sinking speed for the average value of $b_{season}^{\text{model}}$. The former corresponds to *b* ^model^ = 1.110, meaning deeper remineralization and more nutrient accumulation below the pycnocline than the reference run with *b* ^model^ = 1.388 (see details in Methods). Tables 1 and 2 also compare TE_global_ with values for *b* ^model^ = 1.110 and, while it also gives a higher TE in all seasonal scenarios, this seasonal increase is smaller than when $b^{\text{model}}=b_{ref}^{\text{model}}=1.388$ and varies from about 40% to a maximum of 70%.

**Table 1:** Values of TE_global_ for Different Non‐Seasonal Values of *b* ^model^

Constant b^model^ value	0.555	1.110	1.388	2.221
TE_global_	0.33	0.10	0.057	0.011
Relative change to *b* ^model^ = 1.110	+230%	—–	−43%	−89%
Relative change to *b* ^model^ = 1.388	+479%	+75%	—–	−80%

*Note*. All values shown in this table shown are to two significant figures.

**Table 2:** Values of TE_global_ in the Presence of a Global Seasonality of 60% for Different Values of *θ* (in Months)

Seasonally varying b^model^	*θ = 0*	*θ = 3*	*θ = 6*	*θ = 9*
TE_global_	0.14	0.15	0.17	0.16
Change relative to non‐seasonal *b* ^model^ = 1.110	+40%	+50%	+70%	+60%
Change relative to non‐seasonal *b* ^model^ = 1.388	+150%	+160%	+200%	+180%

*Note*. All values shown in this table shown are to two significant figures.

### 3.3 Influence of Seasonality on Primary Production and the Ocean's Biological Carbon Pump

The global effect of *b* ^model^ on PP is summarized in Tables 3 and 4. For the non‐seasonal reference case of *b* ^model^ = 1.388, this model gives a globally integrated PP of around 52 Pg C year^−1^ (56 P C year^−1^ if including areas shallower than 1,080 m too). This value is in agreement with ocean‐observing satellites and other estimates of PP in the literature, which are of approximately 50 Pg C year^−1^, with an uncertainty of 20% (Carr et al., 2006; DeVries & Weber, 2017). In terms of model‐derived estimates, our value is also in agreement with others such as the 48 Pg C year^−1^ reported by Lima et al. (2014).

**Table 3:** Values of PP_global_, *F*_120m_ and *F*_1080m_ (All in Pg C year^−1^) for Different Non‐Seasonal Values of *b* ^model^

Constant b^model^ value	0.555	1.110	1.388	2.221
PP_global_	19.99	43.69	51.63	62.58
Relative change to *b* ^model^ = 1.110	−54.25%	—–	+18.17%	+43.21%
Relative change to *b* ^model^ = 1.388	−61.28%	−15.38%	—–	+21.19%
*F*_120m_	4.85	5.84	5.50	3.97
Relative change to *b* ^model^ = 1.110	−16.95%	—–	−5.82%	−32.02%
Relative change to *b* ^model^ = 1.388	−11.82%	+6.18%	—–	−27.81%
*F*_1080m_	1.59	0.59	0.31	0.040
Relative change to *b* ^model^ = 1.110	+169.49%	—–	−47.46%	−93.22%
Relative change to *b* ^model^ = 1.388	+412.90%	+90.32%	—–	−87.10%

*Note*. All values shown in this table were rounded and therefore the resulting percentiles differ slightly from the ones computed using all significant numbers.

**Table 4:** Values of PP_global_, *F*_120m_ and *F*_1080m_ (All in Pg C year^−1^) in the Presence of a Global Seasonality of 60%, for Different Values of *θ* (in Months)

Seasonally varying b^model^	*θ = 0*	*θ = 3*	*θ = 6*	*θ = 9*
PP_global_	43.61	41.15	37.43	39.82
Change relative to non‐seasonal *b* ^model^ = 1.110	−0.19%	−5.83%	−14.34%	−8.86%
Change relative to non‐seasonal *b* ^model^ = 1.388	−15.53%	−20.30%	−27.50%	−22.87%
*F*_120m_	5.09	5.28	5.30	5.10
Change relative to non‐seasonal *b* ^model^ = 1.110	−12.84%	−9.59%	−9.25%	−12.67%
Change relative to non‐seasonal *b* ^model^ = 1.388	−7.46%	−4.00%	−3.64%	−7.27%
*F*_1080m_	0.69	0.80	0.92	0.81
Change relative to non‐seasonal *b* ^model^ = 1.110	+16.95%	+35.59%	+55.93%	+37.29%
Change relative to non‐seasonal *b* ^model^ = 1.388	+122.58%	+158.07%	+196.77%	+161.29%

*Note*. All values shown in this table were rounded and therefore the resulting percentiles differ slightly from the ones computed using all significant numbers.

The values for PP_global_ in Table 3 (non‐seasonal *b* ^model^) show that the primary production increases with *b* ^model^ and decreases with the sinking speed (see Equation 3). This is expected since a lower *b* ^model^ implies faster sinking, with more nutrient recycling at depth (see Figure 5 for lower values of *b* ^model^), and therefore less nutrient is available for recycling and production. While decreasing *b* ^model^ by 60% (from *b* ^model^ = 1.388 to *b* ^model^ = 0.555) reduces the total PP by 61% to about 20 Pg C year^−1^, an increment of 60% (from *b* ^model^ = 1.388 to *b* ^model^ = 2.221) increases the primary production by only 21% to slightly over 62 Pg C year^−1^.

The introduction of seasonality leads to a very different scenario for productivity. Table 4 shows that, in the case of a seasonal *b* ^model^, there is a consistent decrease in production (and increase in nutrient accumulation, see Figure 5), from about 15% when *θ* = 0 months (43 Pg C year^−1^) to over 27% when *θ* = 6 months (37 Pg C year^−1^). When compared to *b* ^model^ = 1.110, a similar behavior is observed, with the exception of *θ* = 0 months, when PP decreases slightly by a modest 0.2%.

The globally integrated total POC flux in the upper ocean (here defined at the fixed depth of 120 m) is also presented here, integrated over regions greater than 1,080 m in depth (as explained in Methods). Tables 3 and 4 show that it decreases slightly across the seasonal runs, however, we note that this decrease is minimum (maximum) when the decrease in PP is maximum (minimum), which is the scenario shown when *θ* = 6 months (*θ* = 0 months). A similar behavior is shared by the non‐seasonal runs: varying *b* ^model^ from *b* ^model^ = 1.388 to *b* ^model^ = 0.555 and *b* ^model^ = 2.221 results in a decrease of about 11% and 27% respectively; and varying *b* ^model^ from *b* ^model^ = 1.110 to *b* ^model^ = 0.555 and *b* ^model^ = 2.221 results in a decrease of about 16% and 32% respectively. The relative effects shown in Table 3 show an interesting pattern: note that while PP seems to increase with *b* ^model^ and the POC flux at 1,080 m decreases with increasing *b* ^model^, flux at 120 m seems to show signs of both, with a maximum export happening for *b* ^model^ = 1.110.

In the context of carbon sequestering via the ocean's biological pump, we note that the presence of seasonality in *b* ^model^ substantially increases the amount of POC “surviving” the journey through the mesopelagic zone and reaching 1,080 m depth. This amount of POC transferred to the deep ocean is given by *F*_1080m_ and Table 4 shows that, for a seasonal *b* ^model^ with 60% seasonality (i.e., $\delta b=0.6b_{ref}^{\text{model}}$), *F*_1080m_ is increased by at least 119% with respect to the reference case of *b* ^model^ = 1.388, and nearly doubles for *θ* = 6 months. When compared to a run with constant *b* ^model^ = 1.110, there is also a consistent increase in POC transfer for all phases *θ*, but this increase is much less pronounced and capped by about 55%, with the largest increase once again for *θ* = 6 months. However, we note that for *θ* = 0 (meaning maximum *b* ^model^ coincides with maximum solar radiation and PP), only a small increase of less than 17% is observed with respect to the *b* ^model^ = 1.110 run, suggesting that the seasonal variation in *b* ^model^ has a limited effect.

We finally note that these values of *F*_1080m_ observed in the seasonally varying *b* ^model^ simulations are rather different from those observed in the non‐seasonal cases of *b* ^model^ = 0.555 and *b* ^model^ = 2.221, which corresponds to the maximum and minimum values attained by the seasonal *b* ^model^. As shown in Table 3, a 60% lower but constant *b* ^model^ (corresponding to *b* ^model^ = 0.555) increases the POC flux at 1,080 m depth by over 412%, while a 60% higher *b* ^model^ (corresponding to *b* ^model^ = 2.221) actually decreases the flux by about 87%. This distinctive behavior indicates the importance of the various feedback (e.g., changes in nutrient supply at the surfaces due to increased transfer to depth) taking place in the model when *b* ^model^, and consequently the sinking speed, varies throughout the year. In particular, the fact that *F*_1080m_ is consistently higher when *b* ^model^ varies seasonally indicates that, over long time scales (in our case of about 3,000 years), the effects on the BCP due to periods of higher sinking speed (which correspond to periods of small *b* ^model^ and lead to a higher TE) tend to overcome the effects of lower sinking speed periods (which correspond to periods of high *b* ^model^ and lower TE). This might be because, over these time scales, the effect of an increased transfer to depth should be higher if particles entering the deep ocean are trapped there for hundreds to thousands of years, hence becoming unavailable for recycling at the surface for a long period, while the effects of lower transfer would be lower or even null depending on the time of the year.

## 4 Discussion

### 4.1 Seasonal Effects on TE and Productivity

The results presented in the previous section show a broad spectrum of effects on TE as a result of the different relative phase *θ*, which dictates when the flux attenuation is strongest and weakest throughout the year. Even though the seasonally modified *b* ^model^ (see Equation 5) corresponds to a simple cosine curve in all cases, the way it feeds back into the system changes substantially depending when both maximum and minimum are attained, which is controlled solely by the phase *θ*. In particular, the feedback between phase and solar radiation, which dictates the bloom season, explains in part why the largest relative effects on TE and PP happen when *θ* = 6 months. This value of *θ* means a maximum in *b* ^model^ in winter in both hemispheres, which corresponds to the sinking speed being minimum during winter. With productivity being largest during late‐spring, there is more material available on the surface during the summer months, and the coupling with a high sinking speed matching this bloom period maximizes the amount of POC exported to the mesopelagic zone and further down, resulting in a high transfer efficiency. However, this higher transfer efficiency leaves less material available to be recycled back into PO_4_ in the surface, which decreases the amount of nutrient available for primary production, and hence we observe a stronger decrease in PP in this situation. In the model used in this work, this in turn leads to a decrease in phytoplankton and zooplankton, the latter meaning that less detritus is produced (see Kriest & Oschlies, 2015; Kriest et al., 2010). Even with less material available for sinking, the higher efficiency in transporting it to depth increases the absolute amount of POC that survives the journey through the mesopelagic zone and reaches the deep ocean.

**Figure 9:**
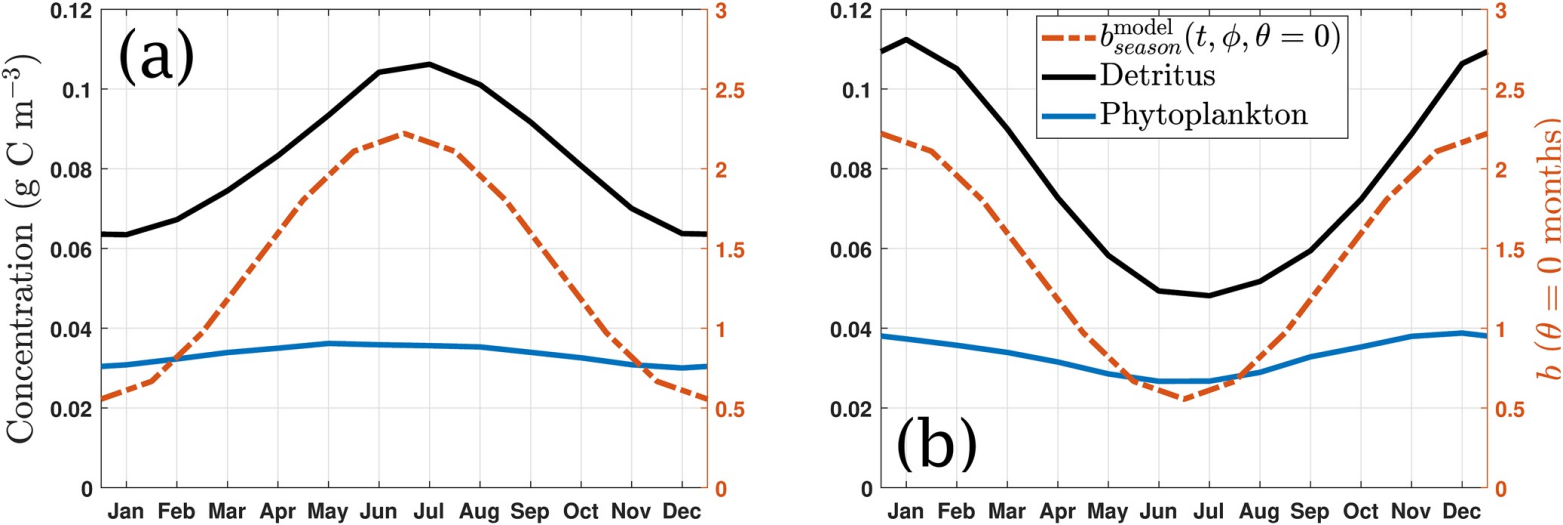
Average surface concentration (g C m^−3^) of detritus (solid black line) and phytoplankton (solid blue line) in the Northern (left, Figure 9a) and Southern (right, Figure 9b) Hemispheres, for the case of constant *b* ^model^ = 1.388. The figure also shows how $b_{season}^{\text{model}}$ (dash‐dotted red line) varies throughout the year in the respective hemisphere, for *θ* = 0 months. Note that the axis on the left shows the concentration and the axis on the right shows the values for *b* ^model^.

In contrast, the effects of seasonality on TE are minimized when *θ* = 0 months (see also Figure 9), where the impact of a variation in the flux attenuation is partially suppressed by both high productivity and detritus production matching periods of higher attenuation and vice‐versa.

The nature of the feedback between *b* ^model^ (and sinking speed), PP and growth can also be seen when looking at the resulting fractional changes in TE, relative to a constant *b* ^model^ = 1.388, for *θ* = 3 and *θ* = 9 months as shown in Figures 8b and 8d respectively. For instance, while in the former we note an increase of over 300% in TE at the Southern Ocean region, the latter displays a 50% decrease in the same areas, with the converse being observed in the subtropical gyres (decrease in TE for *θ* = 3 months and increase for *θ* = 9 months). The asymmetry of these figures also means that having a scenario of fast sinking speed at times of decreasing productivity is not necessarily equivalent to another with slow sinking speed and increasing productivity (see also Section 3.3). This remains true even when the fractional change is measured with respect to a constant *b* ^model^ = 1.110 as shown in the Supporting Information S1.

The heart of the asymmetry seen between *θ* = 3 and *θ* = 9 months lies in the fact that, in the macroscale considered in this study, the intrinsic sinking movement of a POC particle is a unidirectional process (primarily governed by *b* ^model^) and, in our model, has a velocity of order 1/*b* ^model^ (see Equation 4), which is a nonlinear function of *b* ^model^. It also increases linearly with depth, meaning that the speed is slower at the surface and therefore more sensitive to changes. This sinking speed acts on the vertical only and competes with the background ocean circulation, which moves material laterally but also vertically. For small values of *b* ^model^, the POC sinking speed is dominant over the circulation, but for very large *b* ^model^, the circulation might disturb the POC movement, especially at the surface. Hence, a period of very slow sinking speed matching a period of high productivity might not only correspond to the “slow” sinking of abundant surface material but might also carry influence of other processes such as circulation, a scenario that differs from a fast sinking of proportionally less abundant surface material.

Despite the asymmetries between Figures 8b and 8d, a symmetry between these phases is nearly obtained for the global flux *F*_1080m_, as shown in Table 4, indicating that the opposite effects seen in the subtropical gyres and polar areas (e.g., Southern Ocean) tend to balance each other.

### 4.2 Seasonal Variability in TE and Sampling

A direct consequence of a seasonal *b* ^model^ relates to sampling. First, if we consider that *b* is varying seasonally, as suggested by Bol et al. (2018), then when we measure *b* at a place and time we do not know what the phase of the seasonal variation in *b* is relative to PP or export. This means that we do not know whether the *b* measured is the annual maximum, minimum or something in between. As evidenced by the results in Section 3, the phase matters and neglecting it can substantially underestimate or overestimate both TE and carbon fluxes in the ocean when such *b* is used as *b* ^model^.

**Figure 10:**
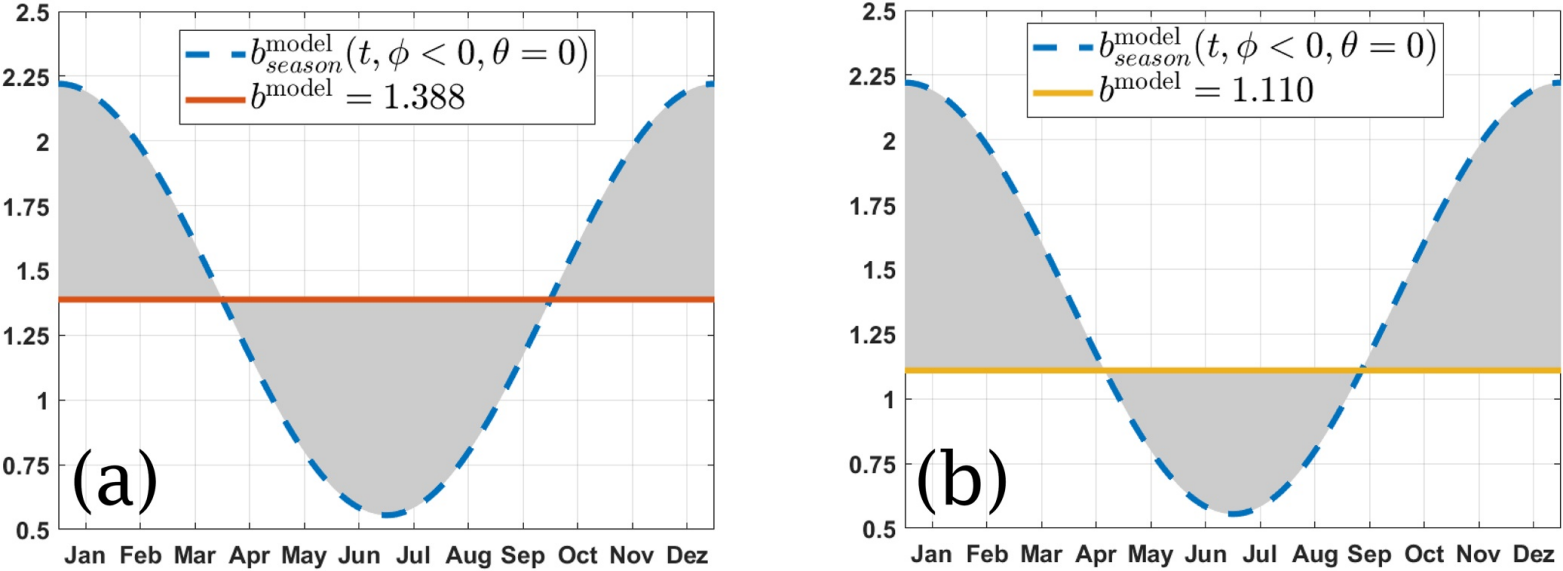
Time variation for $b_{season}^{\text{model}}$ (dash blue line) in the Southern Hemisphere (*ϕ* < 0) compared against two constant values of *b* ^model^, for the case *θ* = 0 months (the same applies when *θ* ≠ 0 and when *ϕ* > 0). When compared to *b* ^model^ = 1.388 (solid red line, left figure), the variation of $b_{season}^{\text{model}}$ is symmetric. If compared to *b* ^model^ = 1.110 (solid yellow line, figure on right), $b_{season}^{\text{model}}$ is no longer symmetric.

Second, as shown in Tables 2 and 4 for *b* ^model^ = 1.388 and *b* ^model^ = 1.110, the results of seasonality are also highly sensitive to the shape of the seasonal curve around the reference value of *b*. This is also schematically indicated in Figure 10, where $b_{season}^{\text{model}}$ is compared against these values of *b* ^model^. Although the model does not reproduce global fields as well with *b* ^model^ = 1.110, this theoretical exercise indicates that, for an extrapolated reference value of *b* (say, the sampled annual mean), the location and value of local extrema (local maximum and minimum) in the annual time series can greatly reduce the potential errors that arise from extrapolated measurements of *b*. In fact, given that most sampling occurs once a year during an interval of 2–3 weeks and at a specific location, having a research team sampling at a specific location during a period of maximum *b* and another team sampling at the same location during a period of minimum *b* would give two very different results. Such studies can be reconciled with an annual time series with enough resolution to evidence the shape of this temporal variability.

Third, results such as Figure 5 and Tables 1, 2, 3, 4 show that running a model with a seasonal *b* ^model^ varying between *b* ^model^ = 0.555 (−60% from *b* ^model^ = 1.388) and *b* ^model^ = 2.221 (+60% from *b* ^model^ = 1.388) gives very different results to simulations with constant *b* ^model^ at either of these extreme values. This means that neglecting the seasonal cycle by assuming that the *b* observed during a research cruise lasting a few weeks is constant all year can lead to significant errors in estimating TE for the whole year. These conclusions are in line with those from Henson et al. (2015), which in the context of export only, observed that any extrapolation of global export obtained from an instantaneous measurement of export efficiency can result in errors of up to ± 60%. As TE may reflect variations in export efficiency, as both are potentially increased by an increase in sinking speed, this error is consequently passed to any TE measurements made in this fashion.

In order to overcome this, it is essential for the community to address how to observe the seasonal pattern of *b* or extract information such as the mean, maximum and minimum values of *b* and its phase relative to other PP and other fields. Autonomous underwater vehicles (AUV), which can collect high temporal and vertical resolution information in remote areas throughout the year (see e.g., Bol et al., 2018; Sanders et al., 2016), offer the possibility to address the lack of information on seasonal variability, though some issues remain, particularly with the measurement of sinking velocities. Additionally, calibration of optical sensors in an AUV may change during the year affecting POC estimates.

### 4.3 Regional Variability in TE and Sampling

In terms of regional variability, we first note that even in the absence of seasonal and spatial variability in the imposed *b* ^model^, strong regional patterns emerge in the diagnosed TE, as shown in Figures 6a and 6b (see also Figures 7a and 7b). These patterns are more pronounced in some regions with a strong circulation such as the Kuroshio and the Gulf Stream. This is also diagnosed by previous work that did not assume a constant flux attenuation such as Weber et al. (2016) (see Figure 5 in their paper), who also observed higher (lower) deep ocean fluxes at high (low) latitudes, in opposition to Henson et al. (2012), where the highest efficiency in POC transfer was observed at lower latitudes.

These results diverge from the expected in two ways. First, if the power law given by Equation 1 is in fact an annually averaged equilibrium solution for detritus fluxes below the euphotic zone, then we should expect that for a constant *b* = *b* ^model^: (a) TE is given by Equation 2; and (b) TE is uniform across the ocean. However, as Figure 6a shows, even in the absence of a seasonal variability in *b* ^model^, strong regional patterns appear in the diagnosed TE. Furthermore, the average value of this diagnosed TE map in Figure 6a is 0.066, while the TE value given by Equation 2 is 0.047.

**Figure 11:**
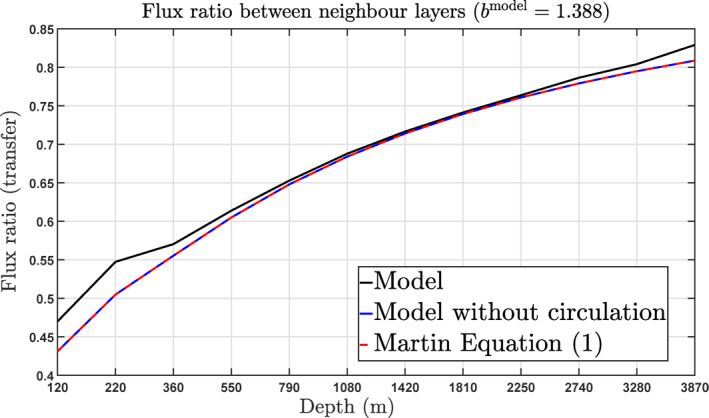
Expected versus diagnosed particulate organic carbon transfer between adjacent layers.

We argue that the main reason for this apparent inconsistency is that both Equation 1 in this paper and Equation (27) in Kriest et al. (2010) do not account for the 3D ocean circulation, which over the course of a year moves material laterally and vertically, and account only the biogeochemical effects on sinking POC in the absence of circulation. In fact, when coupled to the ocean component, extra terms due to circulation are added to the detritus equation, and hence Equation 1 is no longer a solution to this new coupled ocean‐biogeochemistry differential equation. Our analysis shows that the Martin curve given by Equation 1 in general underestimates the POC fluxes and consequently TE. This is also shown schematically in Figure 11, which compares the globally averaged value for the fractional transfer of material that enters layer *k* = 3, …, 14 from layer *k*−1 = 2, …, 13, given by the global average of

$$\frac{\text{Flux}\left(\text{layer}\;k\right)}{\text{Flux}\left(\text{layer}\;k-1\right)},$$

to the values given by Equation 2. The result is that Equation 2 consistently underestimates the fractional transfer between adjacent layers, with a cumulative effect being even stronger and leading to the differences in TE observed for *b* ^model^ = 1.388 discussed above. Note also that this error is larger in the upper layers and decreases in the deep ocean, where the effect of circulation is smaller (while sinking speed is larger). Although some of the difference observed in Figure 11 could be linked to the coarse resolution and limited accuracy of the model, at least some of it should be due to the circulation, giving that switching off the circulation in the model gives a TE map (see Supporting Information S1) that is spatially constant and equals the value of TE predicted by Equation 2. This fact is often neglected, but this analysis suggests that a proper account of the biological carbon pump might have to take the 3D ocean circulation into consideration, particularly on determining how much of this effect we should expect to see in reality.

With the introduction of seasonal variability in *b* ^model^, these spatial patters are highly affected, leading to an even stronger spatial variability signal, as shown in Figure 8 for different phases of *θ*. For instance, if *θ* = 0 months, corresponding to solar irradiation and *b* ^model^ being in phase, we note that TE doubles in the Equatorial and subtropical ocean, but increases more modestly in high latitude areas. For *θ* = 6 months, on the other hand, TE increases everywhere and the higher increase is observed in the high latitude areas, especially in the North Pacific and in the Southern Ocean. As discussed before, a higher transfer to depth is related to the coupling between periods of productivity and export. These also explain in part these regional patterns, since most of the effect is observed at the productive, high latitude areas. However, the roles of both the mixed layer depth and the circulation can also be noted in Figure 8, as the black contour lines highlight some of the major ocean currents such as the Gulf stream (Figure 8d), Antarctic Circumpolar Current (Figure 8c) and Equatorial current (Figure 8a). This signal is likely to emerge from the balance between the POC sinking speed controlled by *b* ^model^, changes in the mixed layer depth, and the 3D ocean circulation which moves POC and other passive tracers around. With both *b* ^model^ (and consequently the sinking speed) and the mixed layer depth changing seasonally, there will be times where the vertical velocity will be dominant (small *b* ^model^) over the ocean circulation and vice versa. Hence, Figure 8 indicates that, in addition to the coupling with PP and growth, both seasonality and phase in *b* ^model^ matter with respect to both the annual ocean circulation and the seasonal changes in the mixed layer depth too.

This suggestion that both circulation and seasonality may lead to spatial patters in the diagnosed *b* (and TE) and could have important consequences for sampling. The geographical pattern seen in Figure 8 indicates that any reasonable fieldwork‐based estimates for *b* depend not only on the time of the year, but also the location where data is being collected. We note that, in this modeling study, the variation in TE at the less seasonal, tropical regions approximates well the global average variation in TE (as per Table 2 and Figures 8a–8d), while the very seasonal high latitudes and subtropical gyres are more sensitive to seasonal variations in *b* ^model^. In fact, for all phases *θ* considered, Figure 8 indicates that the presence of seasonality consistently increases the transfer efficiency by around 150%–200% in the the tropics, a result that is is close to the 145%–198% of global net TE increase range given by Table 2. On the other hand, Figure 8b shows that this increase exceeds 300% in high latitude areas such as the Southern Ocean, and is lower than 50% in the subtropical gyres. Hence, assuming hypothetically that a seasonally varying flux attenuation with *θ* = 3 months represents reality, we have that if (for instance) *b* is estimated from observations made at high latitudes during summer, the resulting value could be much lower than the actual global average. Using such value in a model as the de facto global mean would therefore lead to a globally deeper remineralization and a global increase in transfer efficiency that could diverge significantly from reality. A similar conclusion can be drawn from Figure 8 for *θ* = 0, 6 and 9 months. This comment reflects the importance of moving forward from the constant Martin *b* = 0.858 hypothesis towards a more robust spatial and temporal estimate of *b*. For instance, as in Marsay et al. (2015) and Weber et al. (2016), where spatially varying maps for *b* were derived from the correlation between spatially scattered observations and some property one can estimate globally (such as surface temperature). Similar correlation algorithms, if applied to relevant processes that can be measured at different instants in time (for instance through remote sensing) such as surface ocean circulation and surface temperature, could be successfully implemented to derive seasonally varying maps of *b* and TE.

### 4.4 Impact on Export and Sequestration of Carbon

For the amount of POC leaving the upper ocean (here defined at the fixed depth of 120 m), our model gives, for a constant *b* ^model^ = 1.388, an export of 5.5 Pg C year^−1^ at 120 m, which is higher than the 4 Pg C year^−1^ presented by Henson et al. (2012) but lower than the 6.7 Pg C year^−1^ flux estimated by DeVries and Weber (2017), both for a fixed 100 m. We note here that our model‐based estimate does not include places with water shallower than 1,080 m (see Methods), which excludes the highly productive estuaries, shelves and coastal areas, and therefore it is an underestimated value for export.

Nevertheless, our results show that the sensitivity of POC fluxes to changes in strength and phase of the seasonality directly impact the carbon export and sequestration by the ocean. The values for *F*_120m_ presented in Tables 3 and 4 indicate that a seasonally varying *b* ^model^ tends to decrease the POC export (considered by some authors as the strength of carbon pump, measured as the amount of organic carbon that is exported from the euphotic zone, DeVries & Weber, 2017) by 3%–7% and hence the seasonal values are even closer to the values reported by Henson et al. (2012). The decreased export is a direct consequence of the decrease in PP in the same seasonal runs, as discussed in 4.1.

Our approach ignores differences in the nature of the detritus pool composition, and our results differ from what has been reported by some authors such as Leung et al. (2020), who, in the context of multi‐annual variability, find that a decrease in export production (due to climate change reasons for instance) could be balanced by a negative feedback mechanism in which a shift towards smaller particle size would boost both productivity and export, therefore compensating for the initial predicted decrease. Although this shift was observed in a multi‐annual variability context, there is evidence that similar shifts in community structure and export production may happen seasonally as well, and hence similar feedback could potentially happen throughout a year.

It is worth noting that, for the export, there is currently no consensus on values, with estimates ranging from less than 4 Pg C year^−1^ (Henson et al., 2012) to over 12 Pg C year^−1^ (Laws et al., 2000), with makes it difficult to compare our findings. Another difficulty when comparing these results is the different methodologies used in several of these studies. For example, some estimates of POC export use a fixed export depth, for example, 120 m (such as Henson et al., 2012), while others consider a variable euphotic zone depth (such as Siegel et al., 2014; DeVries & Weber, 2017). In particular, the study of DeVries and Weber (2017) exposes how much these export fluxes can diverge depending on the methodology adopted: while their estimate for an export flux at the variable depth of the euphotic zone is about 9.2 Pg C year^−1^, the flux at fixed 100 m is 6.7 Pg C year^−1^ (i.e., about 38% lower). This is also a difficulty for computational modeling as several models, including the one used in this study, computes only an averaged‐bulk flux at a small number of fixed‐depth values. Hence, it is not entirely a surprise that our estimates are closer to Henson et al. (2012) than to DeVries and Weber (2017). Overcoming this limitation and finding alternatives to the fixed‐depth approach used here is a challenge for a correct measure of the carbon pump strength (Buesseler et al., 2020).

Although a seasonal *b* ^model^ tends to decrease export and consequently reduces the amount of POC available in the mesopelagic zone, it also allows for a more efficient transfer of POC to the deep ocean, for the reasons discussed in Section 4.1. The results from Table 4 show that the seasonality in *b* ^model^ increases the net fluxes at 1,080 m by at least 122%, which leads to a net TE increase of at least 145% when compared to the non‐seasonal case of *b* ^model^ = 1.388 (as per Table 2). Comparing the average amount of POC sequestered in the seasonal case to the non‐seasonal case (see Tables 3 and 4), in which the flux of POC at 1,080 m depth is 0.31 Pg C year^−1^, a seasonal *b* ^model^ would increase the amount of POC sequestered by at least 0.38 Pg C year^−1^. Therefore, neglecting the seasonality in *b* ^model^ when estimating the POC fluxes to depth through models might give at best an underestimated idea of how much carbon is sequestered in reality.

### 4.5 Limitations in This Approach

The approach taken in this study has a few limitations. It uses a low‐resolution physical model and a simplified representation of the ecosystem and biogeochemistry, where the euphotic zone (top 120 m) consists of 2 layers only, with a fixed export depth of 120 m. This low resolution limits the spatial scale on which the complex, dynamic surface processes happen and might hide some second and third order physical and biogeochemical effects, such as eddy formation and variations in the remineralization rate (as a function of temperature for example), that a finer resolution with a higher complexity model could capture. In addition to that, the export depth is fixed at 120 m and, as discussed in Section 4.4, this differs from the real ocean where the export depth can vary substantially and is a major challenge in quantifying the strength of the carbon pump (Buesseler et al., 2020). Nevertheless, this simplified approach has been used to good effect in several biogeochemical studies such as Wilson et al. (2019), Kriest and Oschlies (2013), Niemeyer et al. (2019), and Keller et al. (2016), to cite a few.

This study also considers a remineralization rate *λ* that is constant in time. Although a constant *λ* provides a reasonable large‐scale description of the tracer fields for the non‐seasonal case *b* ^model^ = 1.388 (see e.g., Kriest, 2017; Kriest & Oschlies, 2015), we note that this is not the only choice available when considering a variable sinking speed in such model, since *a*, *b* ^model^ and *λ* are related by Equation 4 (see Section 2). For instance, we could have opted to vary both *λ* and *b* ^model^ seasonally, and in this way, the seasonal variability introduced in the model would impact both sinking speed (through *b* ^model^) and and the rate at which the PO_4_ pool increases (in our model all detritus remineralized necessarily enters the nutrient pool, see Kriest et al., 2010). As a consequence, a seasonally varying *λ* would affect how much nutrient is available to circulate and be recycled in the upper ocean, hence having a potential impact on production and export, and consequently on how much detritus is available to be remineralized. The resulting effects will depend on the relative phase between such seasonally varying *λ*, PP and sinking speed, especially in case *b* ^model^ also varies seasonally (see Supporting Information S1). On the other hand, changes in *b* ^model^ in the model impact the POC sinking speed solely as shown in Equation 4, and therefore it is unlikely that the results obtained in a seasonal *λ* scenario would be always the same as the ones presented in this study. In fact, two cases are tested and presented in the Supporting Information S1. First, if *λ* varies with time and *b* ^model^ is kept constant (in this case *b* ^model^ = 1.388), the result nearly matches the case of a constant *b* ^model^. Second, if both *λ* and *b* ^model^ varies equally, meaning the sinking speed coefficient *a* = *λ*/*b* ^model^ is constant in time, we obtain a scenario that is not comparable to other seasonal cases presented. Despite these caveats, we do not have evidence from real‐world data on whether *λ* varies seasonally nor information on how it could vary to allow for such a choice. In fact, the seasonal variability in temperature shown by the model at each depth in the mesopelagic is mostly below 1 degree Celsius (See Figures S11–S16 in Supporting Information S1) and therefore is unlikely to be able to influence a seasonally varying remineralization rate if a temperature‐dependent remineralization rate was introduced to the model (a similar conclusion holds for a temperature‐dependence in seawater viscosity, see e.g., Taucher et al., 2014). This is opposed to *b* ^model^ where sufficient information is available (see Introduction) and therefore to constrain the seasonal variation to *b* ^model^ is the most robust option available.

The variation imposed on *b* ^model^ is also limited to a cosine perturbation to a globally constant reference value $b_{ref}^{\text{model}}$. It is arguable that a more realistic seasonally varying *b* ^model^, although periodic, might be more irregular because it will arise from seasonal variations in, for instance, oxygen distributions (Cavan et al., 2017) and ecosystem structure (Bach et al., 2019; Ikenoue et al., 2019), which in turn might affect particle size and ballast (Armstrong et al., 2001; Klaas & Archer, 2002). We note that, despite having evidence for seasonal variation in flux attenuation, we do not have a clear picture yet of the pattern of this seasonal variability (see for instance Figure 2 in Mouw et al., 2016). However, in the absence of further information, a cosine‐like *b* ^model^ provides a very simple but effective approach to a theoretical study of the effects of seasonality in such nonlinear system. This provides an indication of potential impact but cannot be taken further until a clearer observational picture is available.

Another limitation of this study is the fact that the time variation in *b* ^model^ is applied instantaneously and uniformly at all depths. Although consistent with the widely adopted steady state assumption in sediment trap sampling (Giering et al., 2017), which is usually justified by the fast sinking speed of up to 1,800 m day^−1^ shown by some marine aggregates such as fecal pellets and marine snow (Giering et al., 2017) which would reach the deep ocean within 1–2 days, this scenario could be seen as unlikely given the slower sinking of other marine snow. Approximating temporal change in sinking speed propagating to a fixed depth by an instantaneous one would impact the particles that are already at deep parts of the ocean in the same way as those that have just entered the mesopelagic zone. To illustrate this, we note that for a constant *b* ^model^ = 1.388, our model gives a constant sinking speed of 4.32 m day^−1^ at 120 m, which increases linearly to 38 m day^−1^ at 1,080 m and to 180 m day^−1^ at 5,000 m. Hence, a particle at 120 m takes about 61 days to go through the mesopelagic zone and further 43 days to get to 5,000 m depth. When a seasonal *b* ^model^ is introduced, these timescales change considerably: at 120 m the sinking velocity in a year varies between 2.70 m day^−1^ (when *b* ^model^ is maximum) and 10.81 m day^−1^ (when *b* ^model^ is minimum), at 1,080 m it varies between 24.31 m day^−1^ (when *b* ^model^ is maximum) to 97.30 m day^−1^ (when *b* ^model^ is minimum) and at 5,000 it varies between 112.56 m day^−1^ (when *b* ^model^ is maximum) to 450.45 m day^−1^ (when *b* ^model^ is minimum). Hence, in the seasonal case, detritus at 120 m would take between 24 (when *b* ^model^ is minimum) to 97 days (when *b* ^model^ is maximum) to reach 1,080 m and between 58 (when *b* ^model^ is minimum) to 235 days (when *b* ^model^ is maximum) to get to a depth of 5,000 m. While this could still be a valid approach to be taken for fast sinking POC, it is likely to cause a bias since our model does not differentiate slow sinking and fast sinking aggregates. To accommodate these complexities and limitations, a conjecture for a more realistic approach would be that the sinking speed and acceleration could be inherited and retained by the particles at surface, so that any changes in *b* ^model^ at surface would be seen at depth only later when the material had sunk. Such mechanism is likely to increase even more the TE with respect to the constant *b* ^model^ case: in the present case, if a period of high productivity coincides with a minimum in *b* ^model^, then there will be initially a fast POC sinking but this will be gradually slowed by an increase in *b* ^model^. If the POC was allowed to “carry” *b* ^model^ with it, then this exported POC would be accelerated at the same “high‐speed” rate, which would increase the amount of POC being exported. The same is true if a high productivity period coincides with a maximum in *b* ^model^, although the effect would be smaller. This increase in POC fluxes to depth might limit even more the nutrient availability at surface waters and decrease both PP and export, but at a much smaller rate as evidenced by Tables 2 and 4.

## 5 Conclusions

This work investigated the effects and the influence that seasonality in both *b* ^model^ and sinking speed has on the global nutrient distributions and carbon fluxes. The presence of seasonality increases TE, independently of the phase, and acts to retain nutrients and carbon in the deep ocean. It is not only the variability of *b* ^model^ that counts, but also the nonlinear feedback effects between the sinking speed and surface primary production and export. This has important consequences for the carbon pump: the sole presence of seasonality was found to increase the flux of POC to 1,080 m by at least 0.38 Pg C year^−1^, a growth of 122% in relation to the non‐seasonal *b* ^model^ = 1.388 flux of 0.31 Pg C year^−1^. An increase in POC flux to 1,080 m is observed even when the comparison is made with the non‐seasonal *b* ^model^ = 1.110 flux and: it increases the flux by least 0.10 Pg C year^−1^, a growth of 17% with respect to the non‐seasonal *b* ^model^ = 1.110 flux of 0.59 Pg C year^−1^.

It is important to point out that the aim of this study was not primarily to reproduce the observed nutrient distributions and carbon fluxes but to examine how these fields may change as a consequence of a currently poorly understood process. One of the consequences of a seasonally varying *b* ^model^ is to move more material to depth ‐ a conclusion that is dependent on the particular parameterization chosen (e.g., Equation 5 in the case of this work), meaning that new observations are needed to unravel the seasonality in POC sinking speed, as well as to further investigate how it relates to variability in TE and deep ocean fluxes. This has also an impact on observational estimates of *b* (as per Equation 1) that are later used in models. While if *b* was constant seasonally, a single observation at a particular location would be representative of the annual mean, for a non‐constant *b* the timing of sampling relative to the seasonal cycle of PP can have a significant impact on inferred annual carbon fluxes and transfer efficiency.

This work leaves some questions unanswered, which can only be addressed by new observations. While the dependence of *b* (used in models as *b* ^model^) on environmental properties such as temperature and phytoplankton size, is increasingly recognised (Marsay et al., 2015; Weber et al., 2016) we still lack observations showing how *b* varies seasonally and co‐varies with these parameters throughout the year. Ideally, we could build up an empirical or mechanistic relation of *b* to those variables, which tend to be easier to estimate and could therefore be used to estimate *b* using observations that can be readily collected throughout the year.

## Supporting information

Supporting Information S1

## Data Availability

The authors used the phosphate climatology data from the World Ocean Atlas (Garcia et al., 2018), freely available at https://www.ncei.noaa.gov/products/world-ocean-atlas, to generate Figure 3b and the “WOA 2018” contour curves shown in Figures 2 and 5. All the other data used in this manuscript and supplementary info was generated by the authors as model output, as described in Section 2, and is freely available at https://www.doi.org/10.5281/zenodo.4926061.
