# Supplementary material for: Influence of Seasonal Variability in Flux Attenuation on Global Organic Carbon Fluxes and Nutrient Distributions
Source: Global Biogeochem Cycles. 2022 Feb 7;36(2):e2021GB007101. doi: 10.1029/2021GB007101 (PMC9286473; doi:10.1029/2021GB007101)
Supplement: Supplementary file 1 — Supporting Information S1 [file GBC-36-0-s001.pdf]

linedepth linedepth

Supporting Information for "Influence of seasonal variability in flux attenuation on global organic carbon fluxes and nutrient distributions"

F. de Melo Viríssimo[1], A. P. Martin[1], and S. A. Henson[1]

[1]National Oceanography Centre, European Way, SO14 3ZH, Southampton, UK

Contents of this file

1. Tables S1 to S2 and Figures S1 to S2: additional tables and figures to the seasonal PP_{global}, total POC fluxes (at 100m and 1,080m) and TE_{global} across all values of θ (phase) and δb (strength) simulated.

2. Table S3 and Figure S4: additional table and figure to illustrate the effect of circulation.

3. Figure S4: additional figures to illustrate the seasonal changes with respect to $b^{model} = 1.110$.

4. Table S4 and Figures S5 to S6: additional table and figures to illustrate the effect of a seasonally varying λ.

5. Figure S7 to S10: larger versions of Figure 5 in the main manuscript.

6. Figure S11 to S16: additional figures showing the 1-year average temperature from the model at different mesopelagic depths (120m to 1,080m) and the maximum deviation from this average throughout the year.

Corresponding author: F. de Melo Viríssimo, National Oceanography Centre, European Way, Southampton, SO14 3ZH, United Kingdom. (f.de.melo.virissimo@noc.ac.uk)

October 1, 2021, 7:11pm

**Seasonally varying b^{model}**	$\theta = \mathbf{0}$	$\theta = \mathbf{3}$	$\theta = \mathbf{6}$	$\theta = \mathbf{9}$
PP_{global}	51.48	50.92	49.88	50.42
Change relative to non-seasonal case $b^{\text{model}} = 1.388$	-0.29%	-1.38%	-3.39%	-2.34%
$F_{120\text{m}}$	5.43	5.52	5.56	5.47
Change relative to non-seasonal case $b^{\text{model}} = 1.388$	-1.27%	+0.36%	+1.09%	-0.55%
$F_{1080\text{m}}$	0.33	0.35	0.38	0.36
Change relative to non-seasonal case $b^{\text{model}} = 1.388$	+6.45%	+12.90%	+22.58%	+16.13%
TE_{global}	0.061	0.064	0.069	0.066
Change relative to non-seasonal case $b^{\text{model}} = 1.388$	+7.02%	+12.28%	+21.05%	+15.79%

Table S1: Values of PP_{global} (Pg C year^{-1}), total POC fluxes (Pg C year^{-1}) at both 120m and 1,080m depth, and TE_{global}, in the presence of a global seasonality of 20% ($\delta b = 0.2 b_{ref}^{\text{model}}$ imposed on $b^{\text{model}} = 1.388$), for different vales of θ (in months).

**Seasonally varying b^{model}**	$\theta = \mathbf{0}$	$\theta = \mathbf{3}$	$\theta = \mathbf{6}$	$\theta = \mathbf{9}$
PP_{global}	49.18	47.85	45.61	46.87
Change relative to non-seasonal case $b^{\text{model}} = 1.388$	-4.75%	-7.32%	-11.66%	-9.22%
$F_{120\text{m}}$	5.34	5.50	5.56	5.40
Change relative to non-seasonal case $b^{\text{model}} = 1.388$	-2.91%	+0.00%	+1.09%	-1.81%
$F_{1080\text{m}}$	0.44	0.50	0.56	0.51
Change relative to non-seasonal case $b^{\text{model}} = 1.388$	+41.94%	+61.29%	+80.65%	+64.52%
TE_{global}	0.082	0.090	0.10	0.095
Change relative to non-seasonal case $b^{\text{model}} = 1.388$	+43.86%	+57.90%	+75.44%	+66.67%

Table S2: Values of PP_{global} (Pg C year^{-1}), total POC fluxes (Pg C year^{-1}) at both 120m and 1,080m depth, and TE_{global}, in the presence of a global seasonality of 40% ($\delta b = 0.4 b_{ref}^{\text{model}}$ imposed on $b^{\text{model}} = 1.388$), for different vales of θ (in months).

(a) $\mathrm{PP_{Global}}$. (b) $\mathrm{F_{120m}}$.

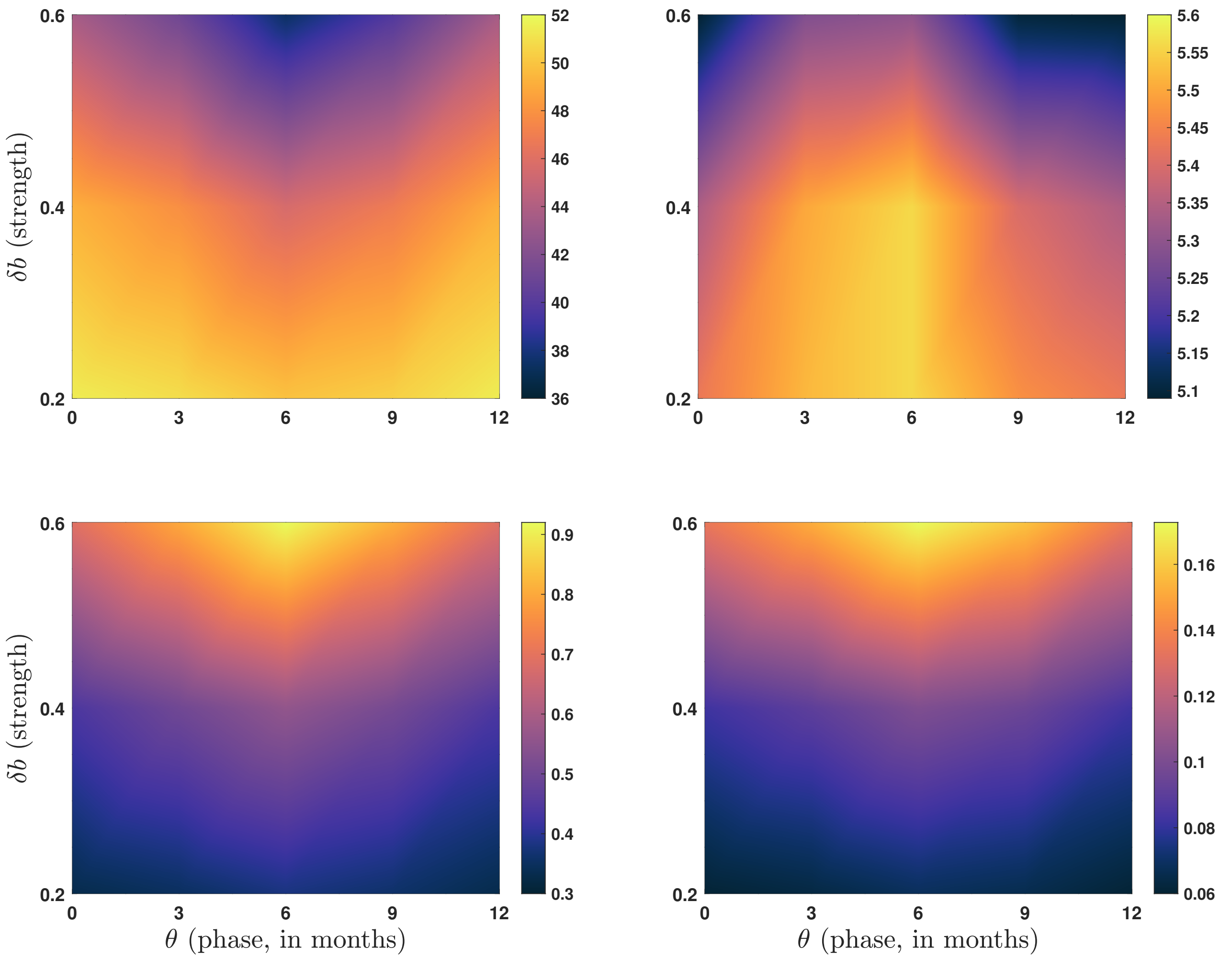

(c) $\mathrm{F_{1080m}}$. (d) $\mathrm{TE_{Global}}$.

Figure S1: Values of $\mathrm{PP_{Global}}$, $\mathrm{F_{120m}}$, $\mathrm{F_{1080m}}$ (all in Pg C year^{-1}) and $\mathrm{TE_{Global}}$, across the range of cases simulated ($\delta b/b_{ref}^{\mathrm{model}}$ from 0.2 to 0.6; and θ from 0 to 12 months) when compared to the non-seasonal case of $b^{\mathrm{model}} = 1.388$. The conclusions are very similar to those already discussed in the main manuscript for $\delta b = 0.6 b_{ref}^{\mathrm{model}}$: the effects of an imposed seasonality are stronger when $\theta = 6$ months and weaker when $\theta = 0$ months for all cases of δb. We also note that these effects are proportional to δb: $\mathrm{PP_{Global}}$ and $\mathrm{F_{120m}}$ decrease when δb increases, while $\mathrm{F_{1080m}}$ and $\mathrm{TE_{Global}}$ increase with δb. Note the different colour bar limits.

(a) $\mathrm{PP}_{\mathrm{Global}}$.

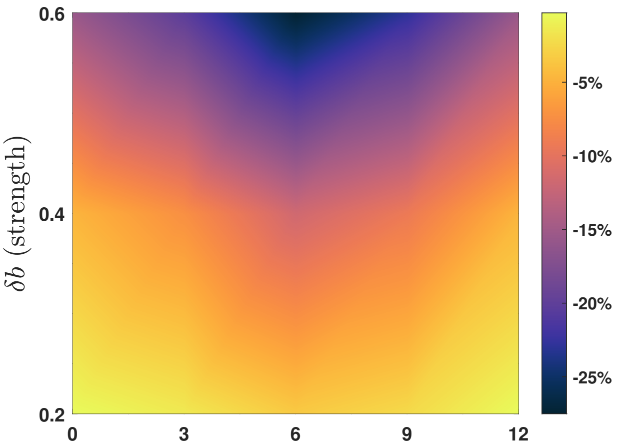

(b) $\mathrm{F}_{120\mathrm{m}}$.

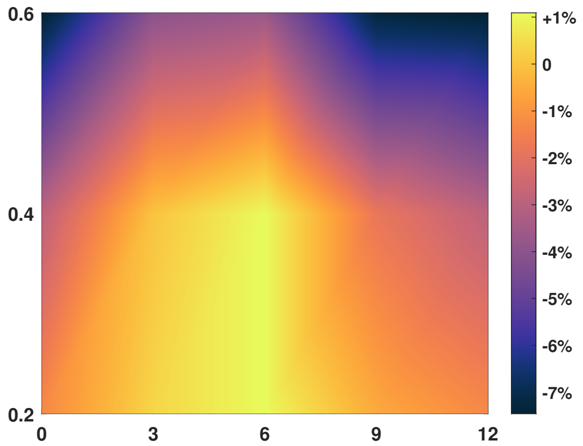

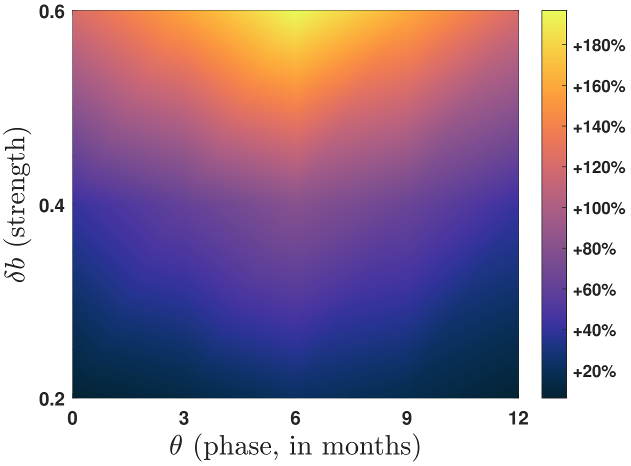

(c) $\mathrm{F}_{1080\mathrm{m}}$.

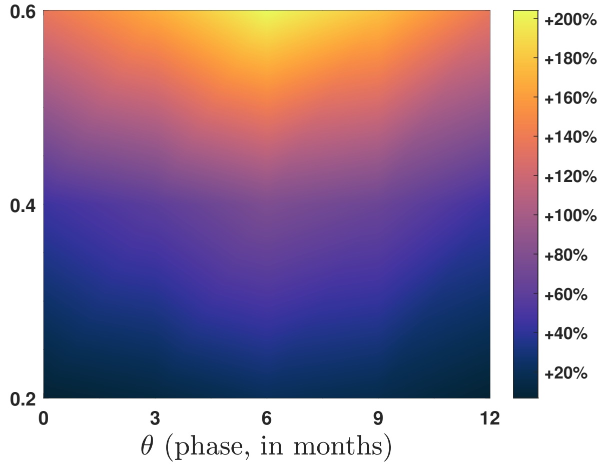

(d) $\mathrm{TE}_{\mathrm{Global}}$.

Figure S2: Percentage change (when compared to the non-seasonal case) in $\mathrm{PP}_{\mathrm{Global}}$, $\mathrm{F}_{120\mathrm{m}}$, $\mathrm{F}_{1080\mathrm{m}}$ and $\mathrm{TE}_{\mathrm{Global}}$, across the range of cases simulated ($\delta b/b_{ref}^{\mathrm{model}}$ from 0.2 to 0.6; and θ from 0 to 12 months) with respect to the non-seasonal case of $b^{\mathrm{model}} = 1.388$. Similar conclusions as in Figure S1. Note the different colour bar limits.

**Non seasonal b^{model} (no circulation on detritus)**	**0.555**	**1.110**	**1.388**	**2.221**
$\text{PP}_{\text{global}}$	20.82	45.63	53.71	64.49
Change relative to circulation case $b^{\text{model}} = 1.388$	-59.68%	-11.62%	**+4.03%**	+24.91%
Change relative to circulation case $b^{\text{model}} = 1.110$	-52.35%	**+4.44%**	+22.93%	+47.61%
$F_{120\text{m}}$	5.18	5.98	5.48	3.72
Change relative to circulation case $b^{\text{model}} = 1.388$	-5.82%	+8.73%	**-0.36%**	-32.36%
Change relative to circulation case $b^{\text{model}} = 1.110$	-11.30%	**+2.40%**	-6.16%	-36.30%
$F_{1080\text{m}}$	1.53	0.52	0.26	0.028
Change relative to circulation case $b^{\text{model}} = 1.388$	+393.55%	+67.74%	**-16.13%**	-90.97%
Change relative to circulation case $b^{\text{model}} = 1.110$	+159.32%	**-11.86%**	-55.93%	-95.25%
$\text{TE}_{\text{global}}$	0.30	0.087	0.047	0.0076
Change relative to circulation case $b^{\text{model}} = 1.388$	+426.32%	+52.63%	**-17.54%**	-86.67%
Change relative to circulation case $b^{\text{model}} = 1.110$	+200.00%	**-13.00%**	-53.00%	-92.40%

Table S3: Values of $\text{PP}_{\text{global}}$ (Pg C year^{-1}), total POC fluxes (Pg C year^{-1}) at both 120m and 1,080m depth, and $\text{TE}_{\text{global}}$ when detritus is not transported by the ocean, for several non-seasonal values of b^{model}. Highlighted in **bold** are the comparison between the circulation and no circulation cases for the same value of b^{model}.

**Seasonally varying λ**	$\lambda_1 \approx b \approx \cos$, $a = $ const.	$\lambda_2 \approx 1/\cos \approx a$, $b = $ const.
$\text{PP}_{\text{global}}$	46.09	51.75
Change relative to $b^{\text{model}} = 1.388$	-10.73%	+0.23%
Change relative to $b^{\text{model}}_{season}(\theta = 0)$	+5.69%	+18.66%
$F_{120\text{m}}$	5.18	5.51
Change relative to $b^{\text{model}} = 1.388$	-5.82%	+0.18%
Change relative to $b^{\text{model}}_{season}(\theta = 0)$	+1.77%	+8.25%
$F_{1080\text{m}}$	0.59	0.31
Change relative to $b^{\text{model}} = 1.388$	+90.32%	+0.00%
Change relative to $b^{\text{model}}_{season}(\theta = 0)$	-14.49%	-55.07%
$\text{TE}_{\text{global}}$	0.11	0.057
Change relative to $b^{\text{model}} = 1.388$	+92.98%	+0.00%
Change relative to $b^{\text{model}}_{season}(\theta = 0)$	-21.43%	-59.29%

Table S4: Values of $\text{PP}_{\text{global}}$ (Pg C year^{-1}), total POC fluxes (Pg C year^{-1}) at both 120m and 1,080m depth, and $\text{TE}_{\text{global}}$ in the presence of a global seasonality of 60% ($\delta b = 0.6 b^{\text{model}}_{ref}$) for different scenarios of λ. In scenario 1, we have $\lambda_1(t) = (\lambda_{ref}/b^{\text{model}}_{ref}) b^{\text{model}}_{season}(\theta = 0)$ and $b^{\text{model}} = b^{\text{model}}_{season}(\theta = 0)$, meaning that the sinking speed coefficient $a = \lambda_1/b^{\text{model}} = \lambda_{ref}/b^{\text{model}}_{ref} = 0.05/1.388 = $ constant. In scenario 2, we have $\lambda_2(t) = (\lambda_{ref} b^{\text{model}}_{ref})/b^{\text{model}}_{season}(\theta = 0)$ and $b^{\text{model}} = b^{\text{model}}_{ref} = 1.388$, meaning that the sinking speed coefficient $a = \lambda_2/b^{\text{model}} = \lambda_{ref}/b^{\text{model}}_{season}(\theta = 0)$.

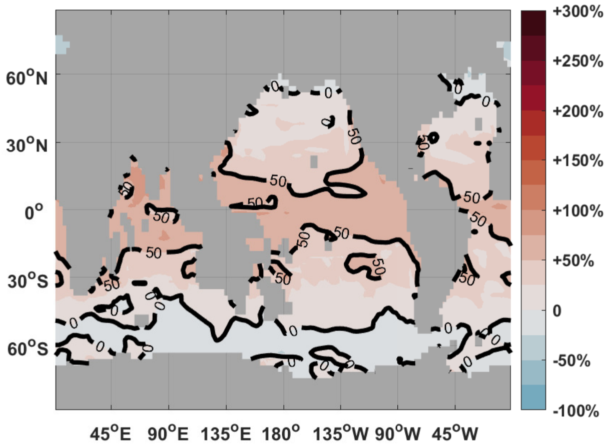

(a) $\theta = 0$ months.

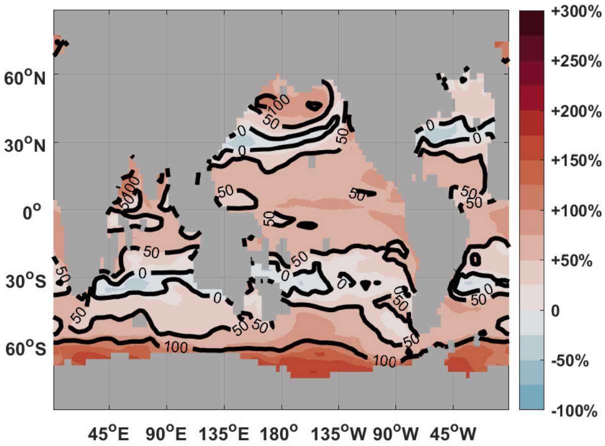

(b) $\theta = 3$ months.

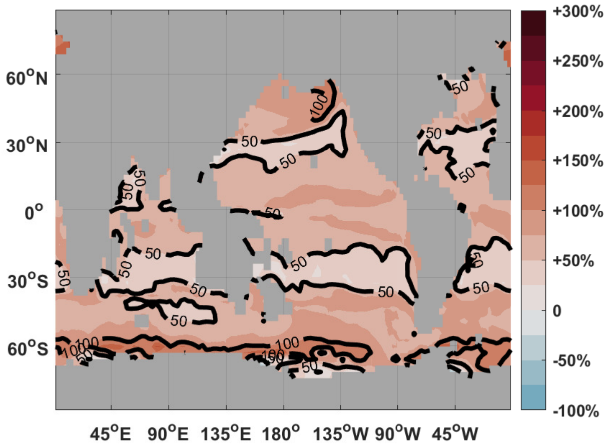

(c) $\theta = 6$ months.

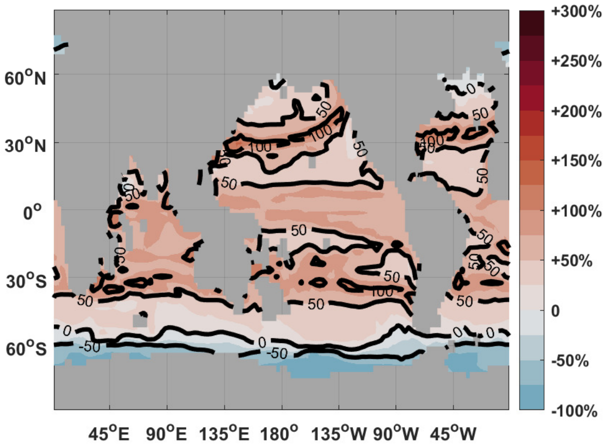

(d) $\theta = 9$ months.

Figure S3: Percentage change (when compared to the non-seasonal case) in annual transfer efficiency (TE) for the parameterisation $b^{\text{model}}_{season}$ with $\delta b = 0.6 b^{\text{model}}_{ref}$, when compared to the non-seasonal case of $b^{\text{model}} = 1.388$. Some of the darker red areas of high-TE have a TE increase in excess of 150% in some locations such as south of $60°S$ in (b).

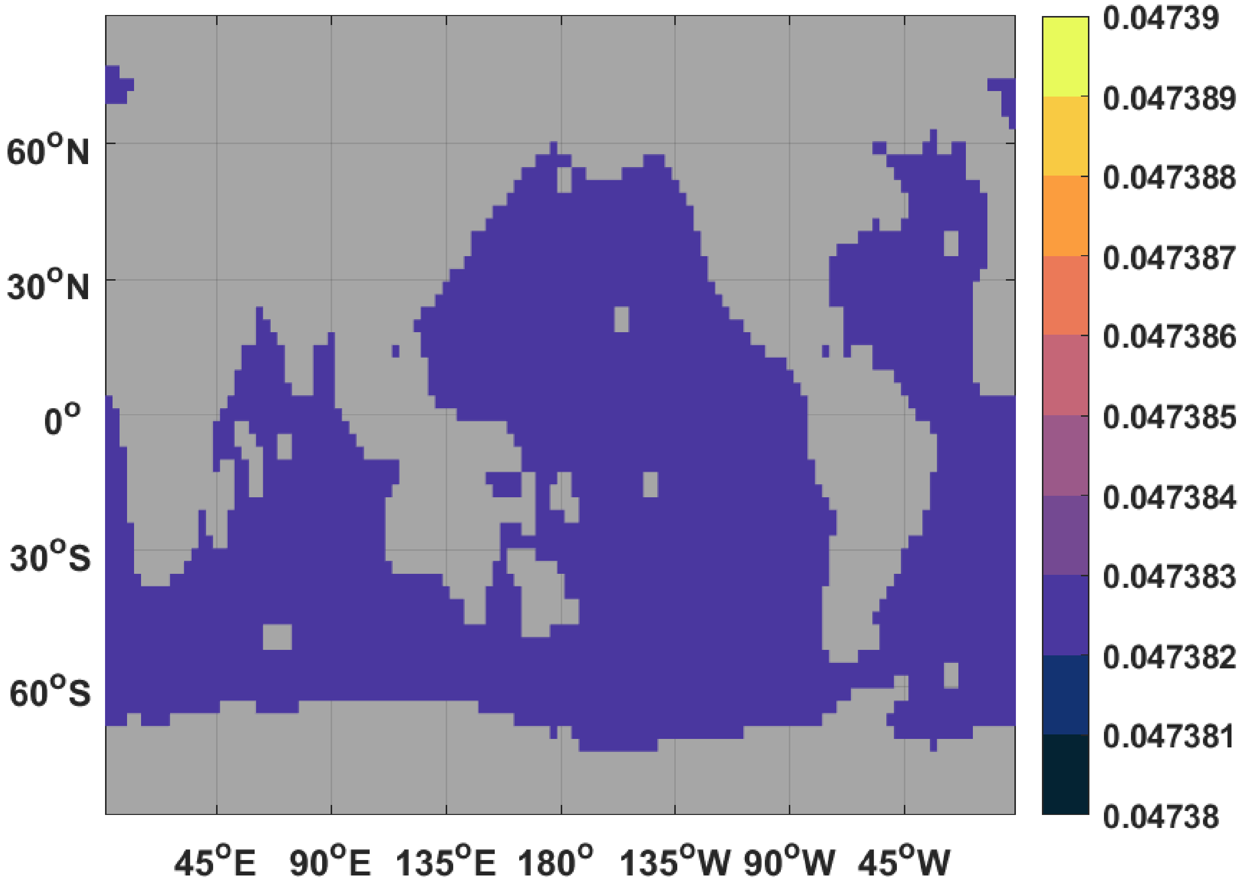

(a) TE for $b^{\mathrm{model}} = 1.388$ without circulation effects on detritus.

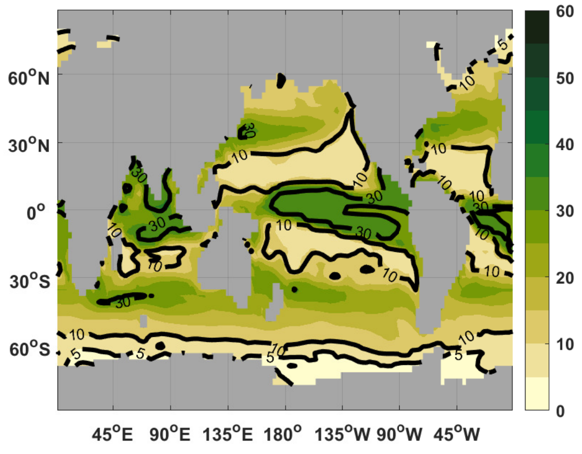

(b) 1-year average POC fluxes at 120m.

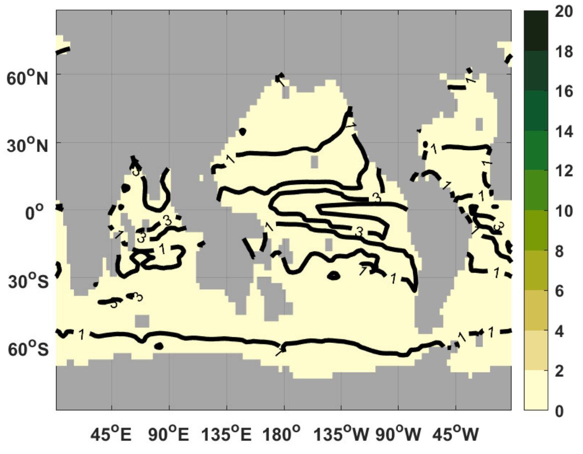

(c) 1-year average POC fluxes at 1080m.

Figure S4: 1-year average POC transfer efficiency (TE) and fluxes (g C m^{-2} year^{-1}) at both 120m and 1,080m depth, for the case of constant $b^{\mathrm{model}} = b^{\mathrm{model}}_{ref} = 1.388$ with detritus not being moved by the ocean circulation. Note the different colour bar limits in plots b and c.

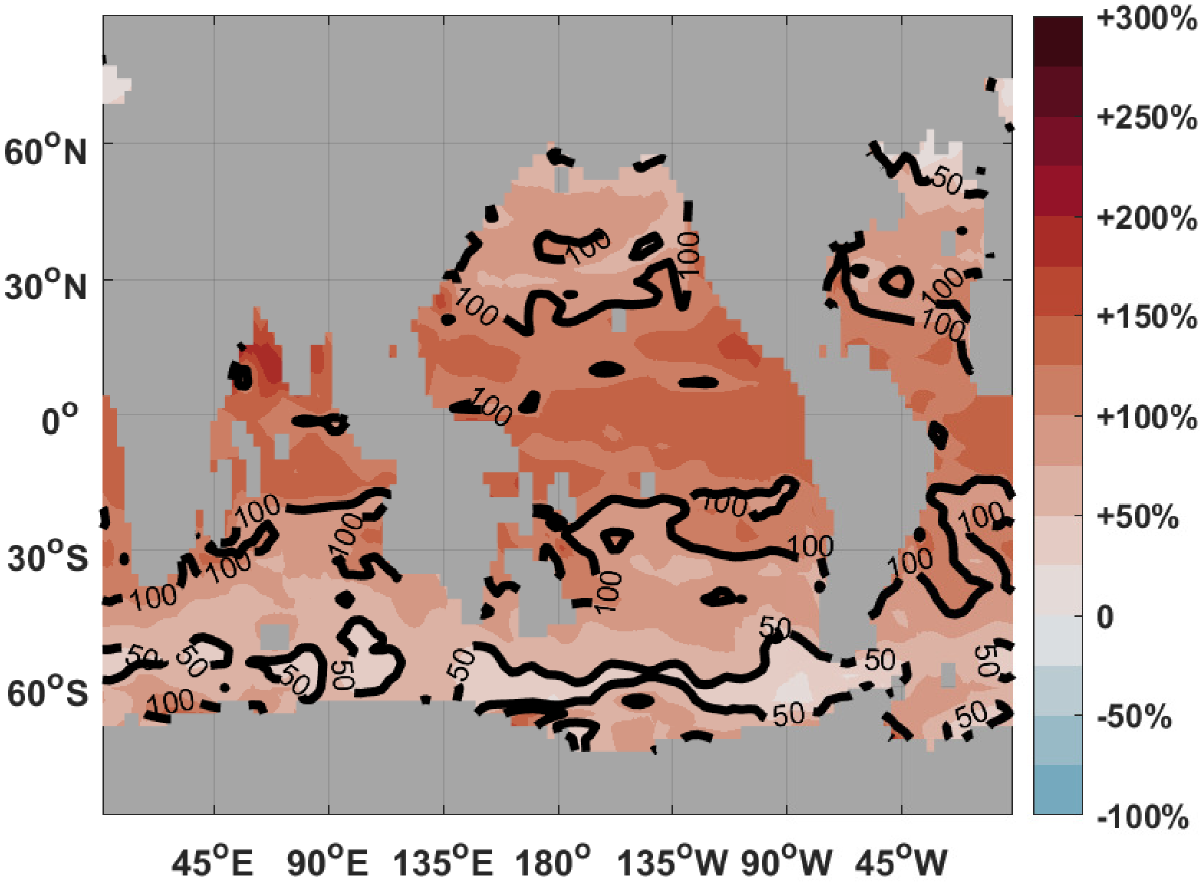

Figure S5: TE changes relative to non-seasonal case of $b^{\text{model}} = b^{\text{model}}_{ref} = 1.388$, for $\lambda_1(t) = (\lambda_{ref}/b^{\text{model}}_{ref})b^{\text{model}}_{season}(\theta = 0)$ and $b^{\text{model}} = b^{\text{model}}_{season}(\theta = 0)$, meaning that the sinking speed coefficient $a = \lambda_1/b^{\text{model}} = \lambda_{ref}/b^{\text{model}}_{ref} = 0.05/1.388 = \text{constant}$.

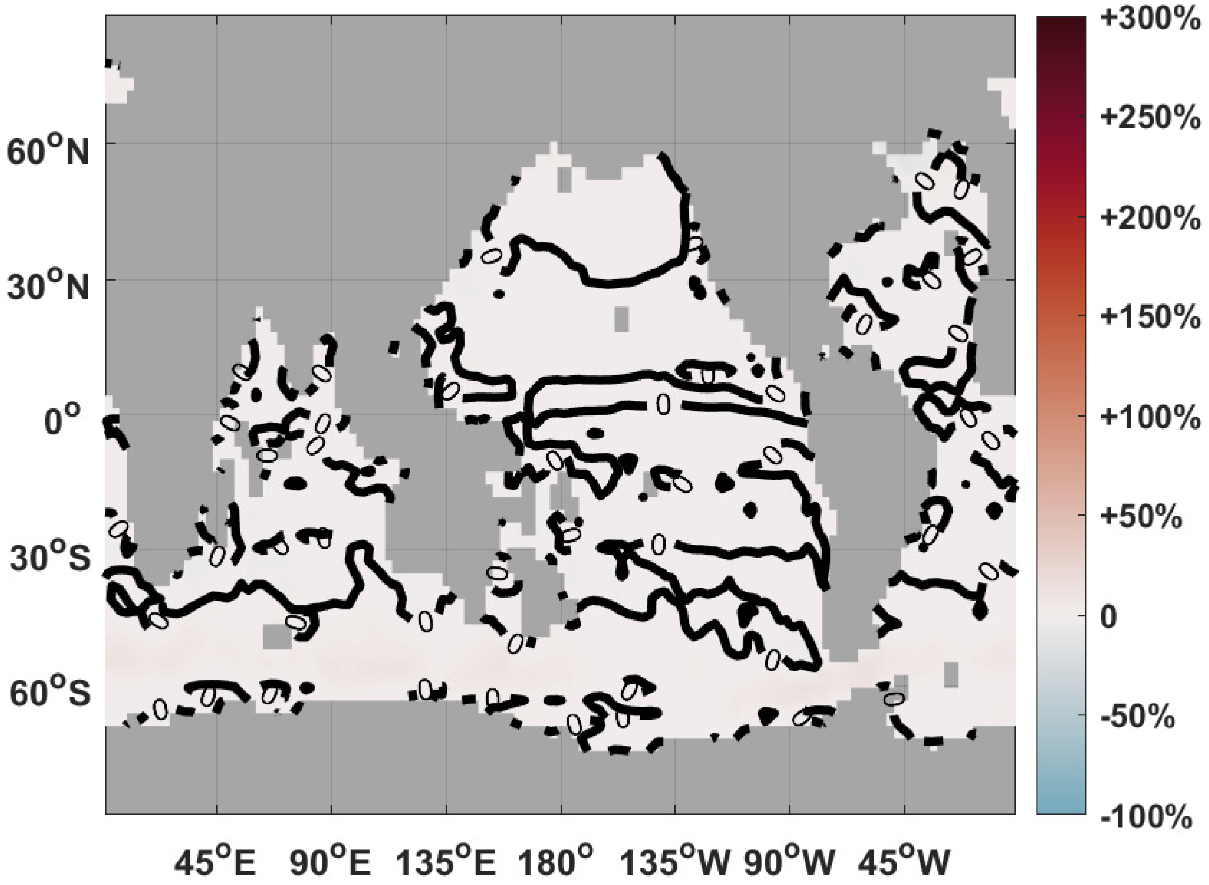

Figure S6: TE changes relative to non-seasonal case of $b^{\text{model}} = b^{\text{model}}_{ref} = 1.388$, for $\lambda_2(t) = (\lambda_{ref} b^{\text{model}}_{ref})/b^{\text{model}}_{season}(\theta = 0)$ and $b^{\text{model}} = b^{\text{model}}_{ref} = 1.388$, meaning that the sinking speed coefficient $a = \lambda_2/b^{\text{model}} = \lambda_{ref}/b^{\text{model}}_{season}(\theta = 0)$.

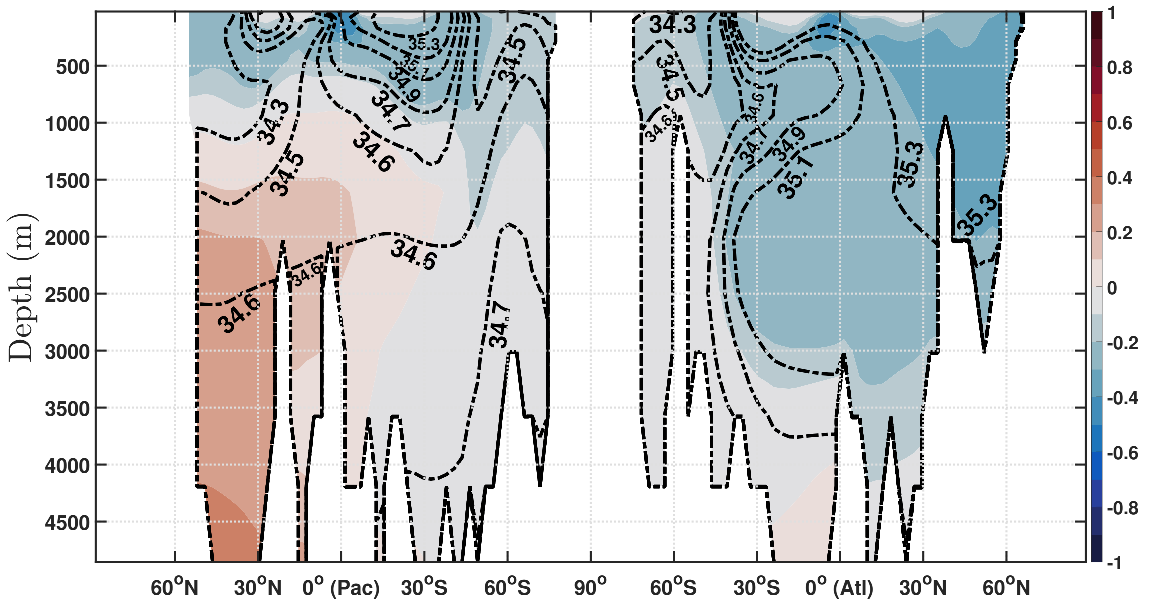

(a) $\theta = 0$ months, with salinity contour lines superimposed.

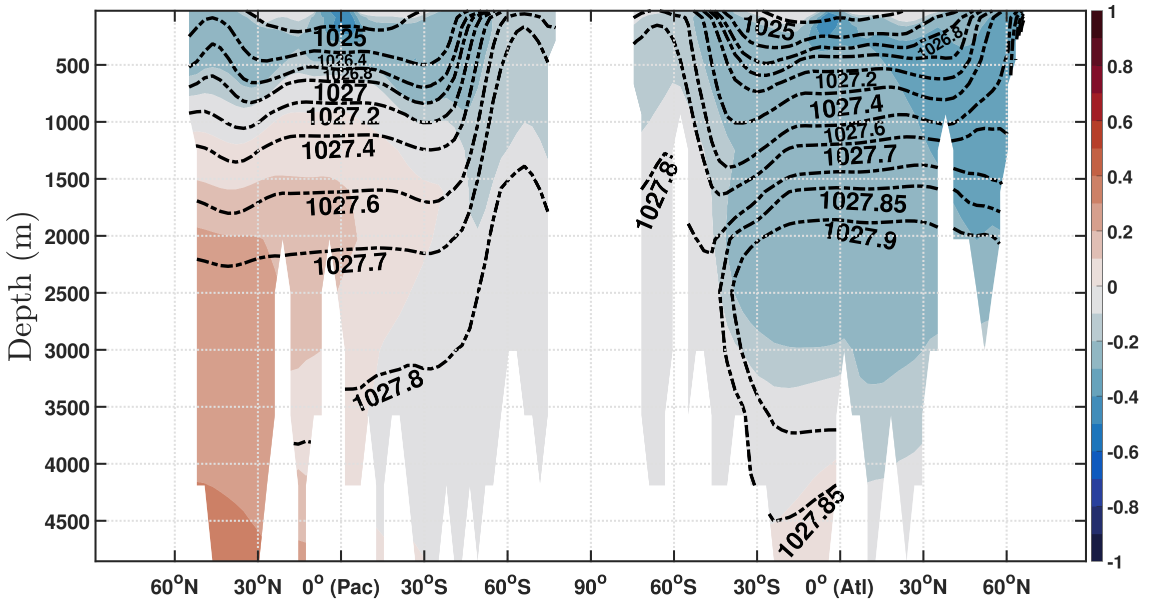

(b) $\theta = 0$ months, with density contour lines superimposed.

Figure S7: Changes in the concentrations (mmol P m^{-3}) for a cross-section of the global ocean (Pacific on the left, Atlantic on the right) for $b^{\mathrm{model}}_{season}$ with a seasonality of 60% and $\theta = 0$ months, when compared to the non-seasonal case shown in Figure 3a in the main manuscript.

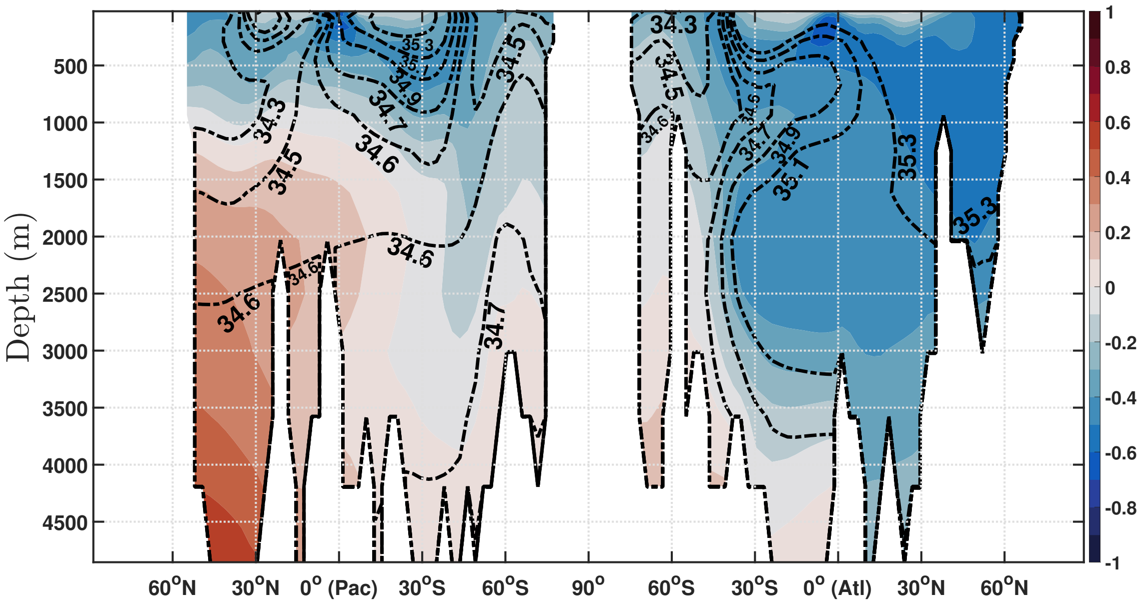

(a) $\theta = 3$ months, with salinity contour lines superimposed.

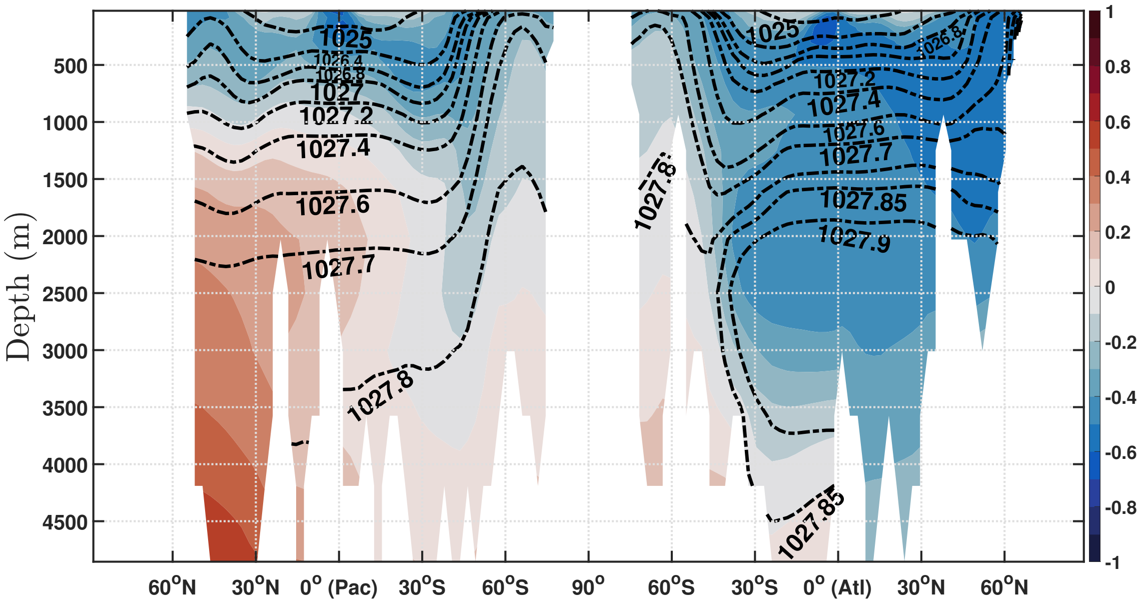

(b) $\theta = 3$ months, with density contour lines superimposed.

Figure S8: Changes in the concentrations (mmol P m^{-3}) for a cross-section of the global ocean (Pacific on the left, Atlantic on the right) for $b^{\text{model}}_{season}$ with a seasonality of 60% and $\theta = 3$ months, when compared to the non-seasonal case shown in Figure 3a in the main manuscript.

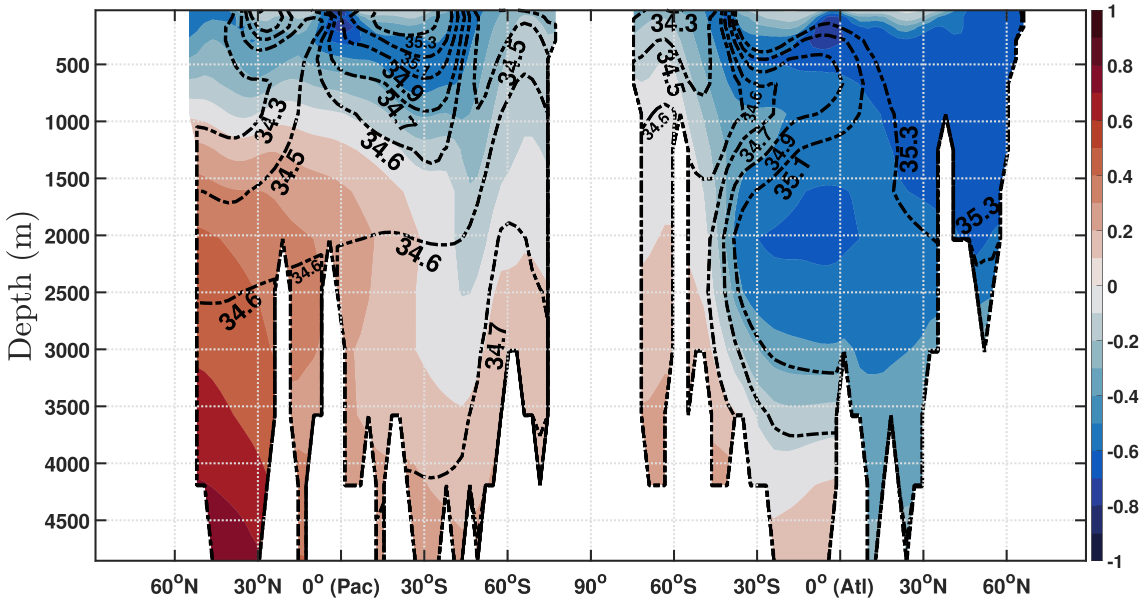

(a) $\theta = 6$ months, with salinity contour lines superimposed.

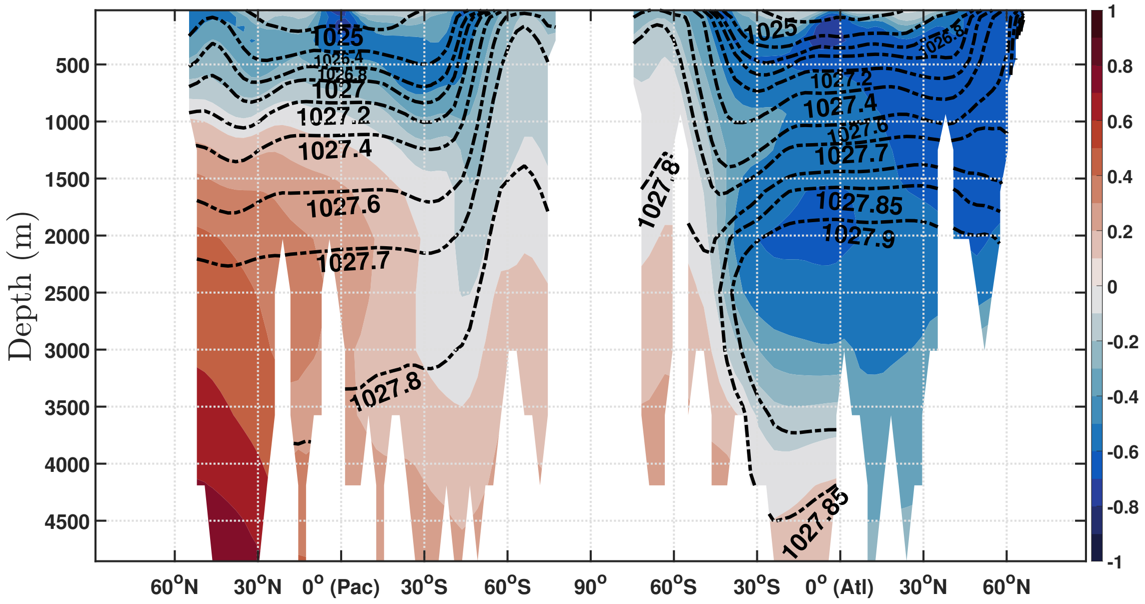

(b) $\theta = 6$ months, with density contour lines superimposed.

Figure S9: Changes in the concentrations (mmol P m^{-3}) for a cross-section of the global ocean (Pacific on the left, Atlantic on the right) for $b^{\mathrm{model}}_{season}$ with a seasonality of 60% and $\theta = 6$ months, when compared to the non-seasonal case shown in Figure 3a in the main manuscript.

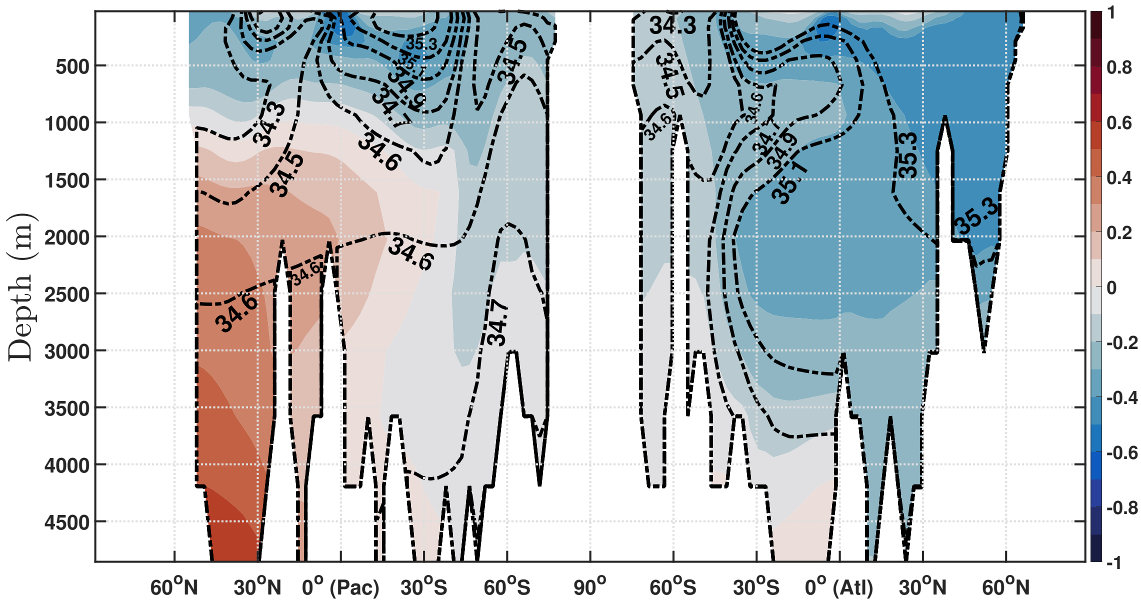

(a) $\theta = 9$ months, with salinity contour lines superimposed.

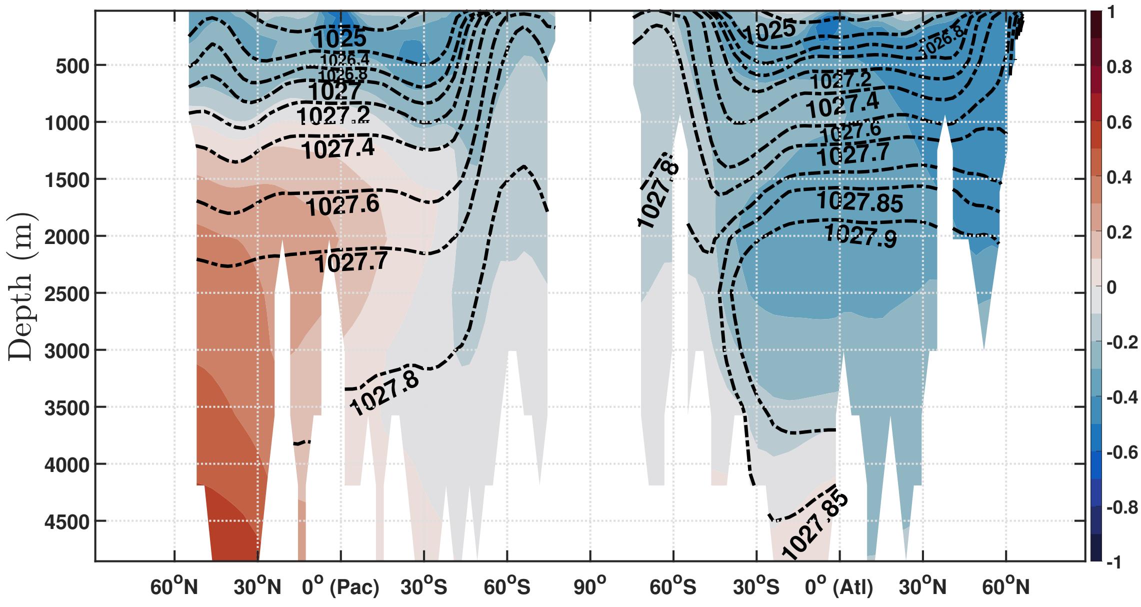

(b) $\theta = 9$ months, with density contour lines superimposed.

Figure S10: Changes in the concentrations (mmol P m^{-3}) for a cross-section of the global ocean (Pacific on the left, Atlantic on the right) for $b_{season}^{\mathrm{model}}$ with a seasonality of 60% and $\theta = 9$ months, when compared to the non-seasonal case shown in Figure 3a in the main manuscript.

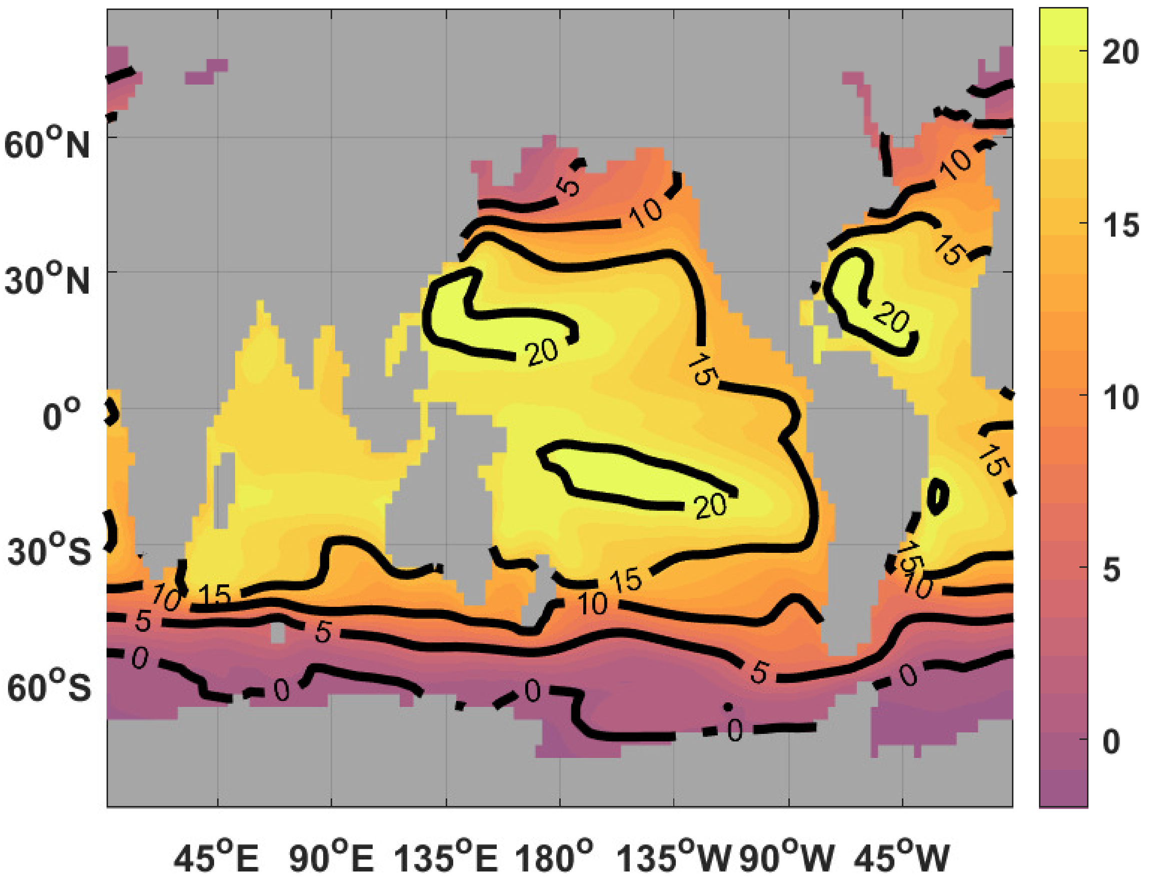

(a) 1-year mean temperature (in degrees Celsius) at 120m.

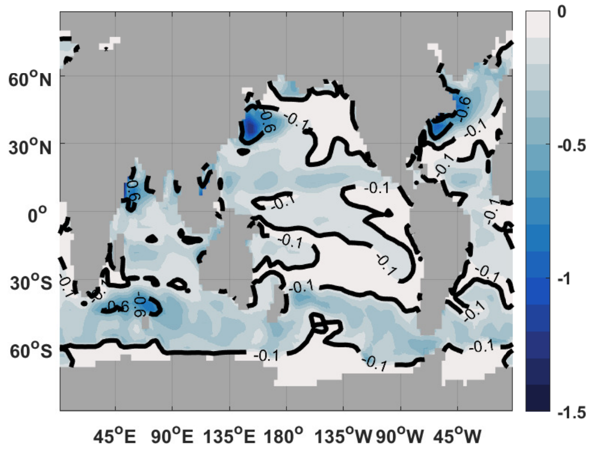

(b) Maximum decrease from mean at 120m.

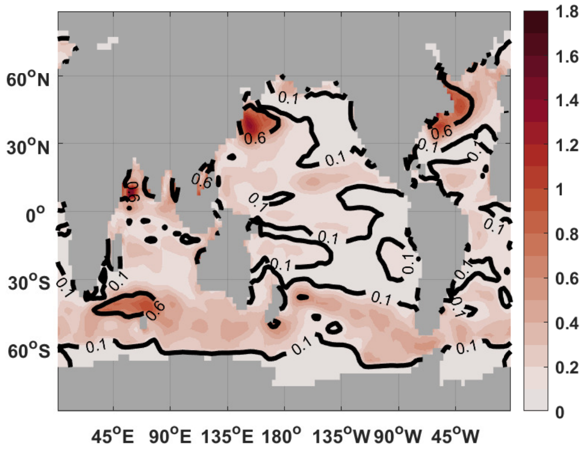

(c) Maximum increase from mean at 120m.

Figure S11: 1-year mean temperature (in degrees Celsius) at 120m from the MITgcm 2.8deg ocean circulation model used in this work. Figures (b) and (c) shows respectively how much this mean temperature decreases and increases (in degrees Celsius) throughout the year. Note the different colour bar limits in all plots.

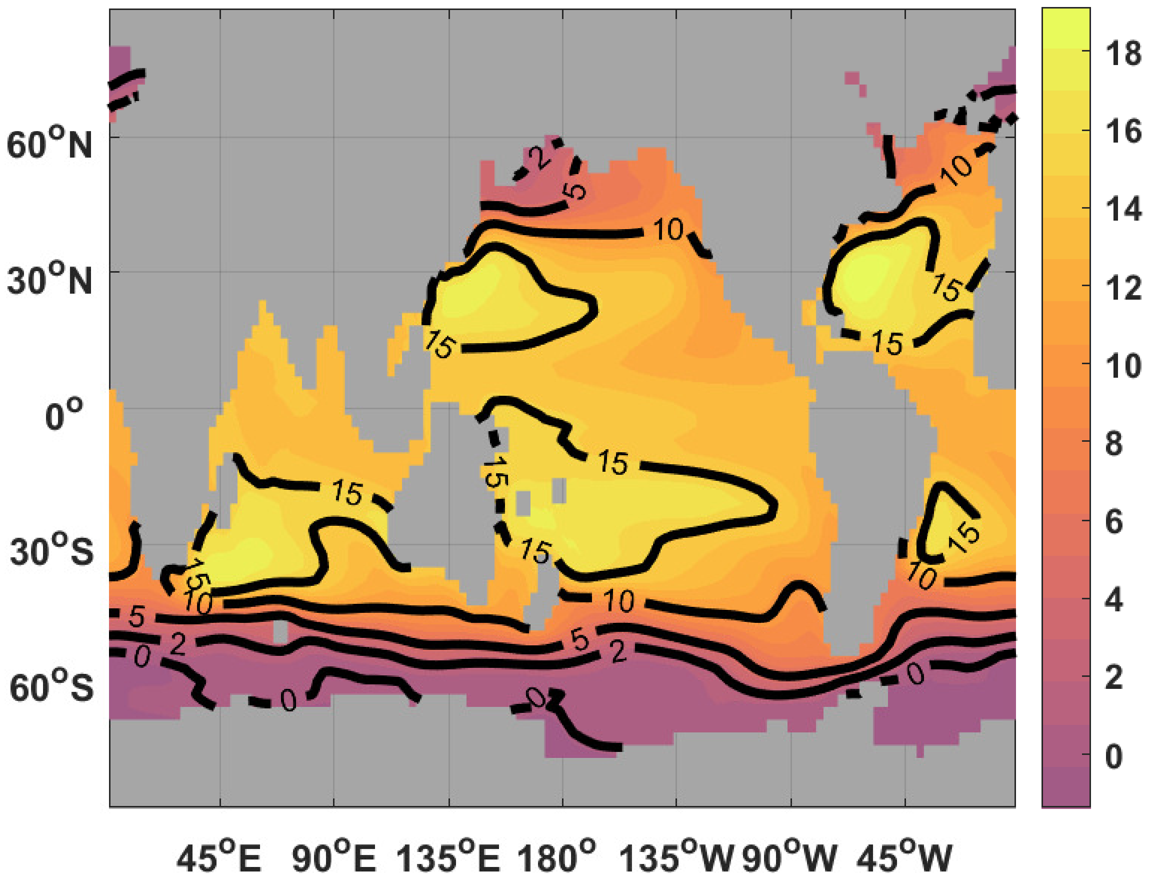

(a) 1-year mean temperature (in degrees Celsius) at 220m.

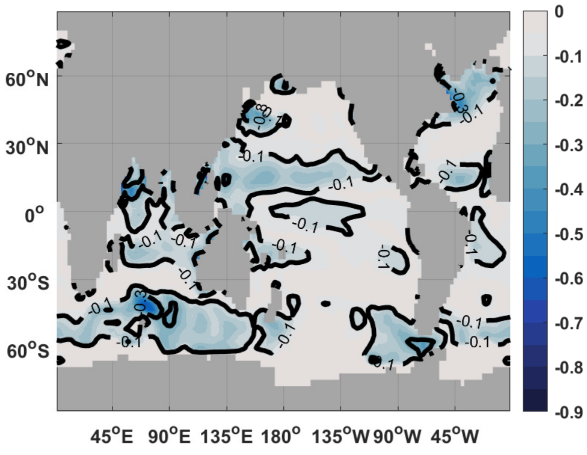

(b) Maximum decrease from mean at 220m.

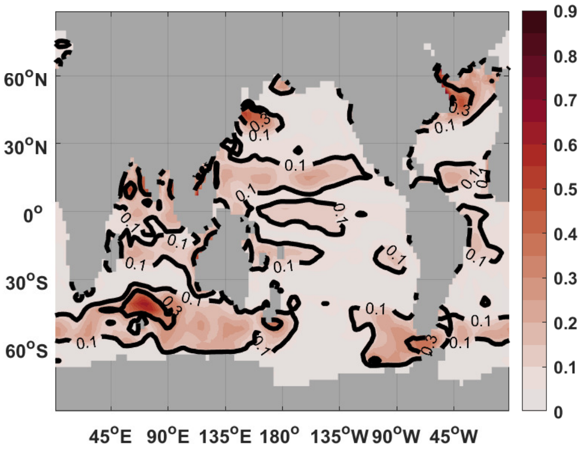

(c) Maximum increase from mean at 220m.

Figure S12: 1-year mean temperature (in degrees Celsius) at 220m from the MITgcm 2.8deg ocean circulation model used in this work. Figures (b) and (c) shows respectively how much this mean temperature decreases and increases (in degrees Celsius) throughout the year. Note the different colour bar limits in all plots.

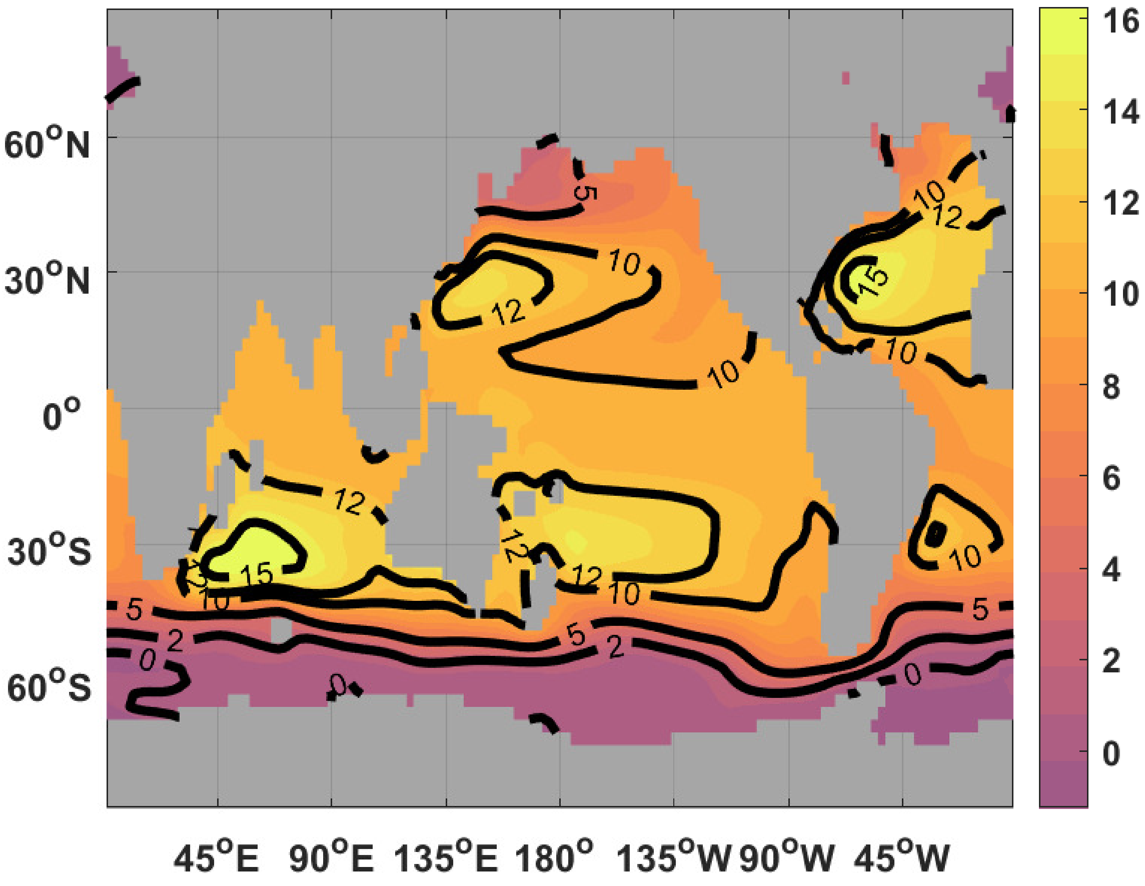

(a) 1-year mean temperature (in degrees Celsius) at 360m.

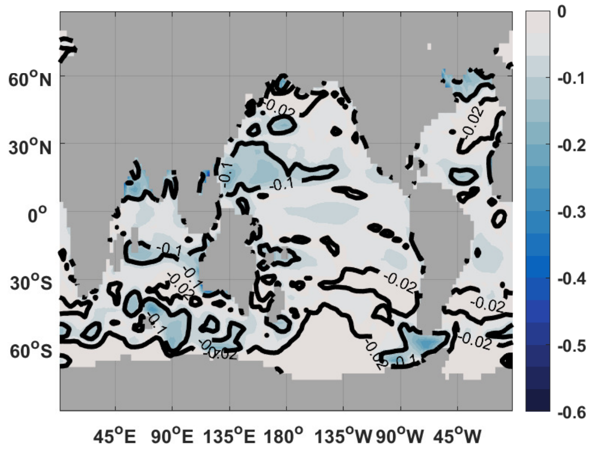

(b) Maximum decrease from mean at 360m.

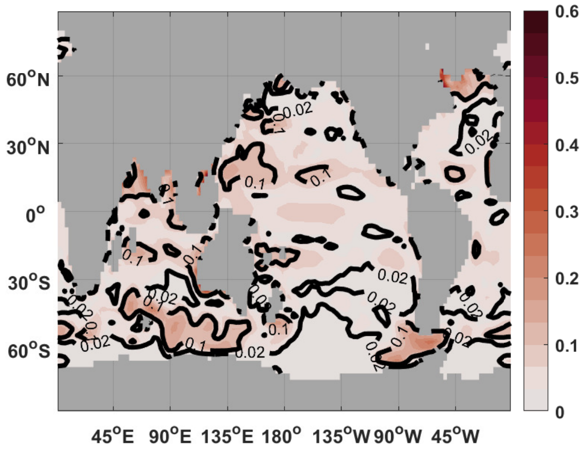

(c) Maximum increase from mean at 360m.

Figure S13: 1-year mean temperature (in degrees Celsius) at 360m from the MITgcm 2.8deg ocean circulation model used in this work. Figures (b) and (c) shows respectively how much this mean temperature decreases and increases (in degrees Celsius) throughout the year. Note the different colour bar limits in all plots.

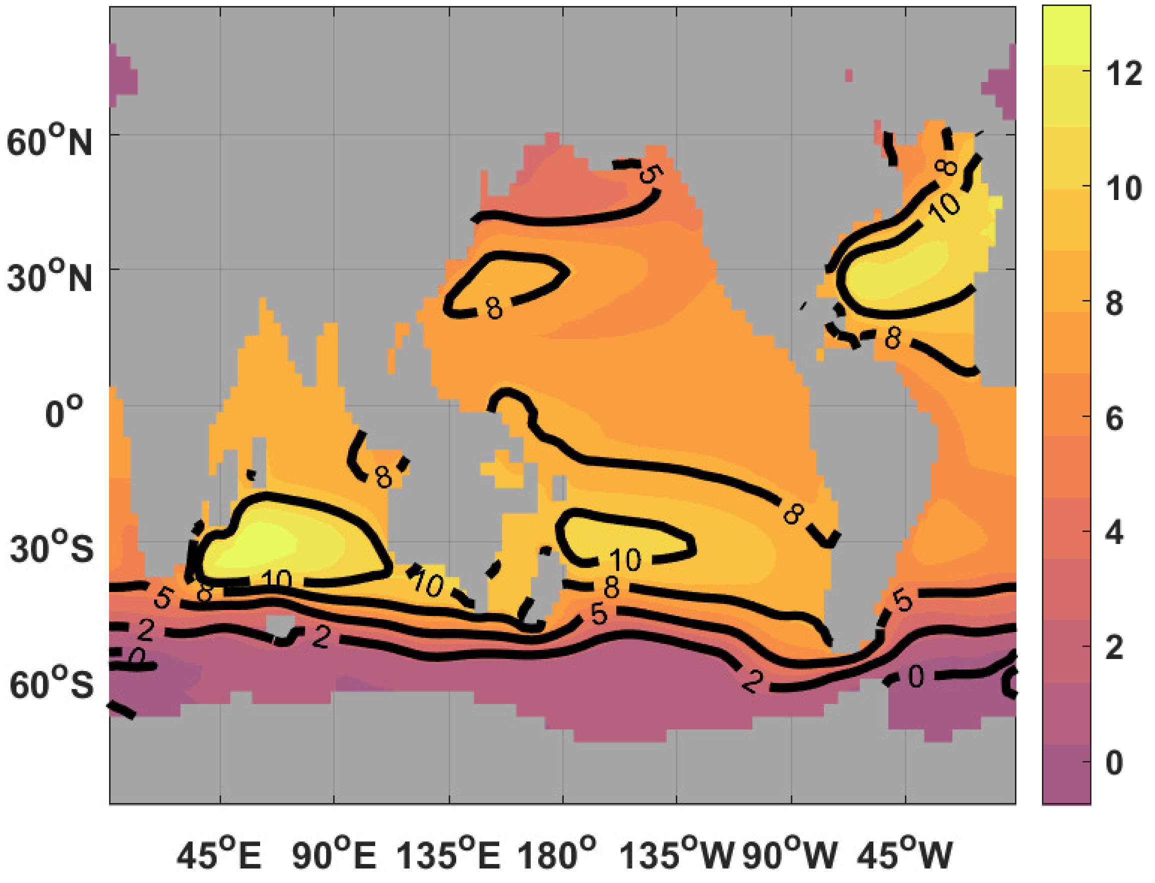

(a) 1-year mean temperature (in degrees Celsius) at 550m.

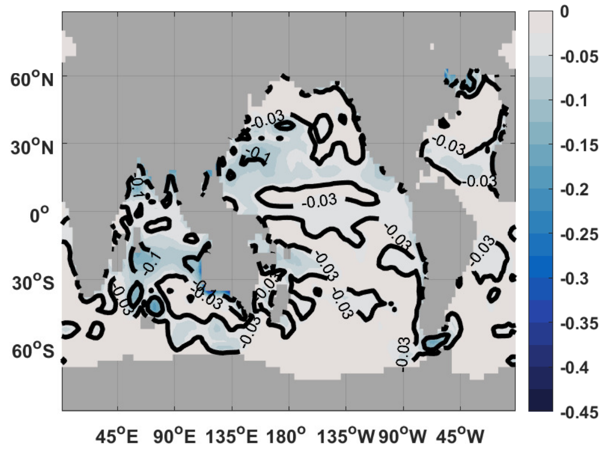

(b) Maximum decrease from mean at 550m.

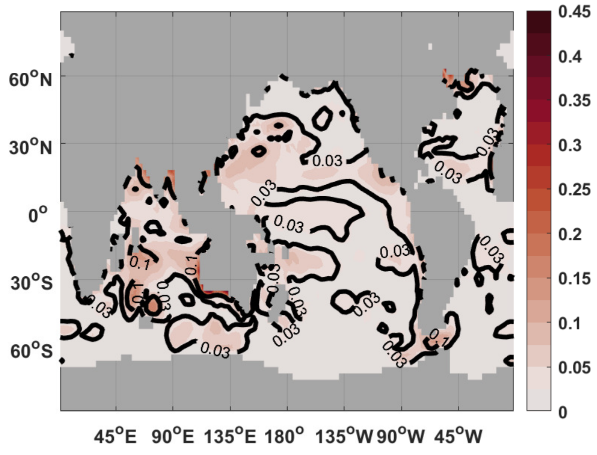

(c) Maximum increase from mean at 550m.

Figure S14: 1-year mean temperature (in degrees Celsius) at 550m from the MITgcm 2.8deg ocean circulation model used in this work. Figures (b) and (c) shows respectively how much this mean temperature decreases and increases (in degrees Celsius) throughout the year. Note the different colour bar limits in all plots.

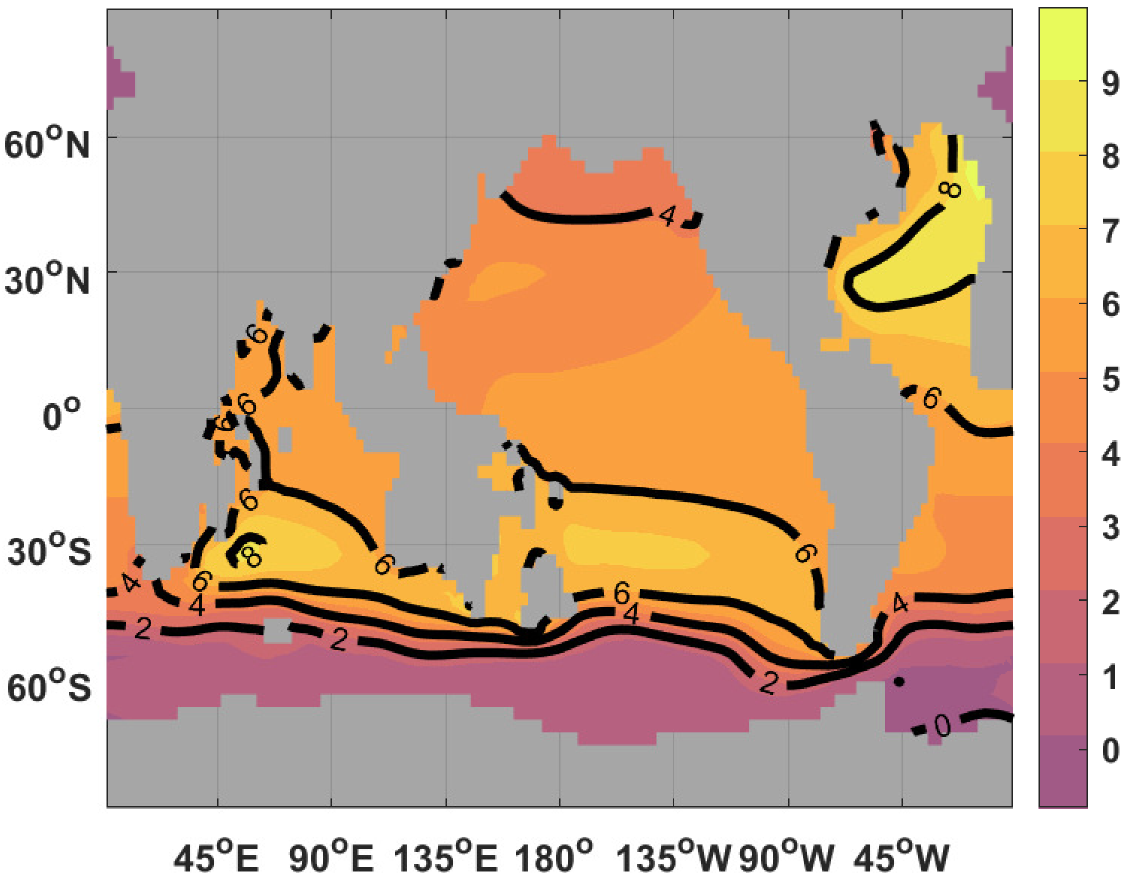

(a) 1-year mean temperature (in degrees Celsius) at 790m.

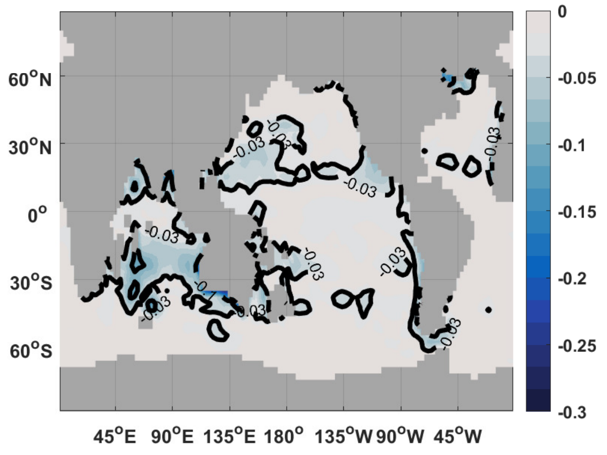

(b) Maximum decrease from mean at 790m.

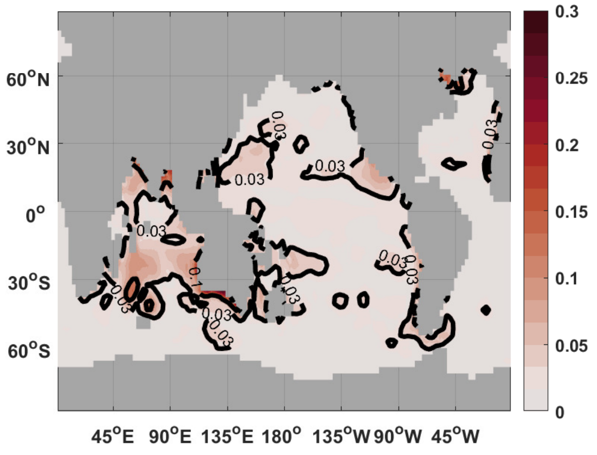

(c) Maximum increase from mean at 790m.

Figure S15: 1-year mean temperature (in degrees Celsius) at 790m from the MITgcm 2.8deg ocean circulation model used in this work. Figures (b) and (c) shows respectively how much this mean temperature decreases and increases (in degrees Celsius) throughout the year. Note the different colour bar limits in all plots.

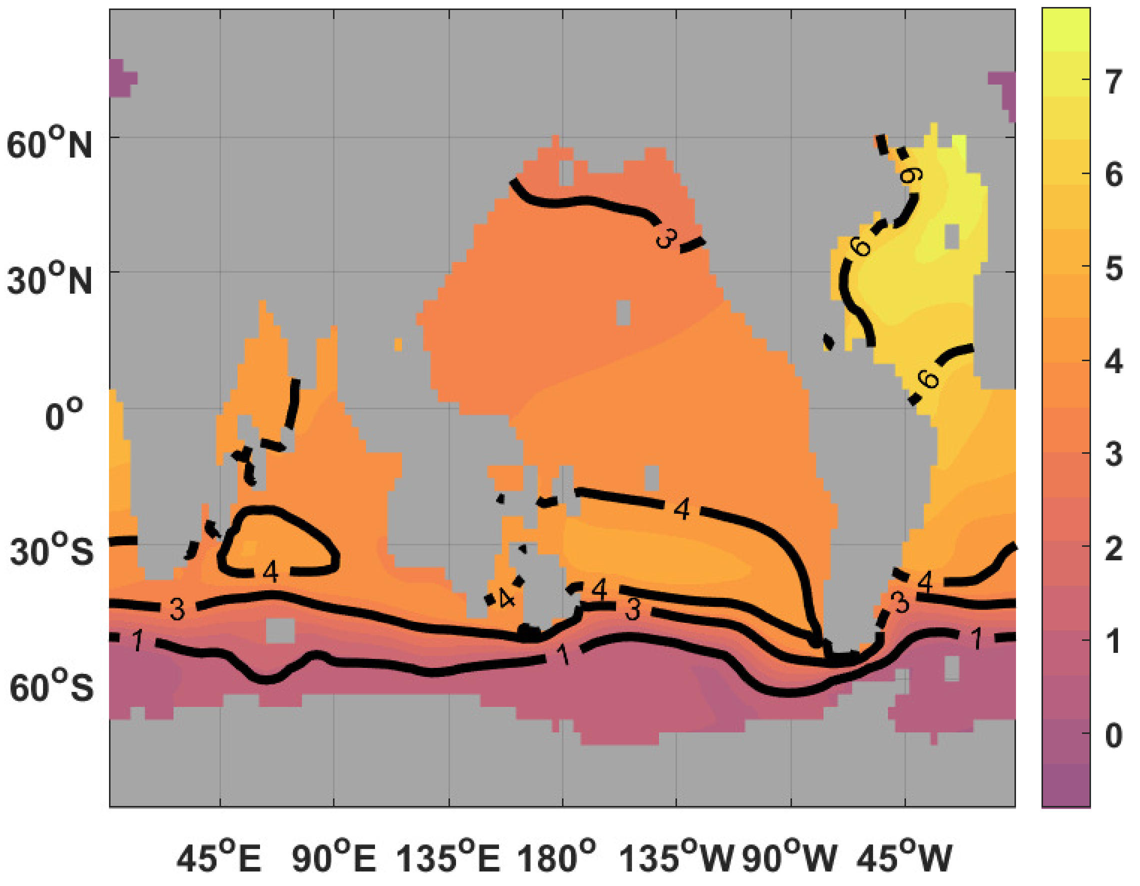

(a) 1-year mean temperature (in degrees Celsius) at 1,080m.

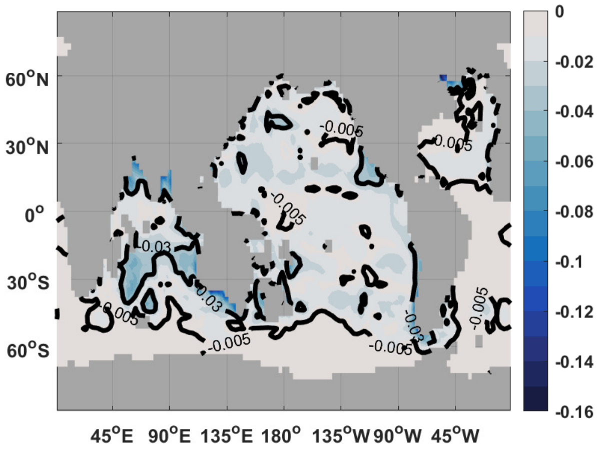

(b) Maximum decrease from mean at 1,080m.

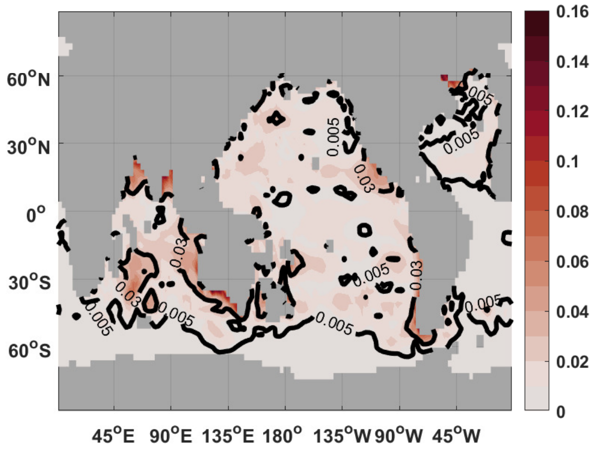

(c) Maximum increase from mean at 1,080m.

Figure S16: 1-year mean temperature (in degrees Celsius) at 1,080m from the MITgcm 2.8deg ocean circulation model used in this work. Figures (b) and (c) shows respectively how much this mean temperature decreases and increases (in degrees Celsius) throughout the year. Note the different colour bar limits in all plots.
